# HERV‐K TM Subunit Elicits CD8^+^ T Cell Anergy and Tumor Immune Evasion via Targeting CD3 Coreceptor ε in AML and PDAC

**DOI:** 10.1002/advs.202417432

**Published:** 2025-08-13

**Authors:** Mengyuan Li, Shuwen Zheng, Qinyuan Gong, Zhaoxing Wu, Wen Lei, Wanyue Cao, Ping Wang, Xuzhao Zhang, Wenbin Qian, Yun Liang, Ying Lu, Fenglin Li, Qi Zhang, Rongzhen Xu

**Affiliations:** ^1^ Department of Hematology and Cancer Institute (Key Laboratory of Cancer Prevention and Intervention, China National Ministry of Education) the Second Affiliated Hospital Zhejiang University School of Medicine Hangzhou 310009 China; ^2^ Institute of Hematology Zhejiang University Hangzhou 310009 China; ^3^ Department of Hepatobiliary and Pancreatic Surgery The First Affiliated Hospital Zhejiang University School of Medicine Hangzhou 310009 China; ^4^ Zhejiang Provincial Key Laboratory of Pancreatic Disease The First Affiliated Hospital Zhejiang University School of Medicine Hangzhou 310009 China; ^5^ MOE Joint International Research Laboratory of Pancreatic Diseases The First Affiliated Hospital Zhejiang University School of Medicine Hangzhou 310009 China; ^6^ Department of Hematology The Affiliated People's Hospital of Ningbo University Ningbo 315000 China

**Keywords:** AML, CD3ε, CD8^+^ T cell anergy, HERV‐K, immune evasion, PDAC, VICs

## Abstract

CD8^+^ T cell anergy is a critical driver of cancer immune evasion, but the underlying causes and mechanisms remain elusive. Here, the functional human endogenous retroviruses‐K envelope (HERV‐K Env) subunit transmembrane (K‐TM) is identified as a potent viral immune checkpoint that induces CD8^+^ T cell anergy and elicits immune evasion in acute myeloid leukemia (AML) and pancreatic duct adenocarcinoma (PDAC). K‐TM subunits are highly expressed in CD8^+^ T cells and enriched in sera of cancer patients. K‐TM‐low CD8^+^ T cells show potent tumor‐killing ability, whereas K‐TM‐high CD8^+^ T cells are incapable of eliciting anti‐tumor effects. Both intracellular and extracellular K‐TM inhibit CD8^+^ T cell activation and cytokine release, leading to CD8^+^ T cell anergy. Mechanistically, K‐TM directly binds to the ITAM domain of CD3ε receptor via its transmembrane domain (TMD), inhibiting CD3ε phosphorylation and disabling TCR signaling. In mouse models, K‐TM reduces CD8^+^ T cell infiltration in tumor tissues and elicits immune evasion. Targeting K‐TM reverses CD8^+^ T cell anergy, restores T cell‐mediated tumor cell killing and regresses PDAC in animal model. The findings for the first time define viral immune checkpoint K‐TM subunit as potent driving force of immune evasion and represent a conceptually new target for immune therapies.

## Graphical abstract

Novel viral immune checkpoint K‐TM subunit is aberrantly expressed in CD8^+^ T cells and enriched in sera of cancer patients, inducing CD8^+^ T cell anergy and eliciting potent tumor immune escape in mouse model. Mechanistically, K‐TM directly targets and induces the phosphorylation of CD3ε receptor. Targeting K‐TM improves CD8^+^ T cell anti‐tumor efficacy in vitro and in vivo.

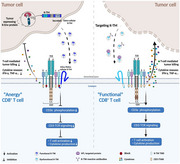

## 1 Introduction

Although great progress has been made in cancer immunotherapies, only a small part of patients respond to this treatment.^[^ ^1^, ^2^, ^3^, ^4^ ^]^ A major challenge to eradicate cancer is the development of immune evasion, primarily in dysfunction of T cells that are critical in antitumor immunity.^[^ ^5^, ^6^, ^7^, ^8^, ^9^, ^10^, ^11^ ^]^ In cancer patients, T cells, especially CD8^+^ T cells, are frequently incapable of eliciting antitumor responses, namely “anergy,”^[^ ^12^, ^13^, ^14^, ^15^, ^16^ ^]^ but the cause and mechanism largely remains unknown. We and others previously observed HERV‐K envelope carrying TM subunit with potential immunosuppressive activity (IS) are preferentially enriched in tumor tissues and closely associated with poor outcomes in a variety of cancers, such as acute myeloid leukemia (AML), pancreatic duct adenocarcinoma (PDAC), lung cancer, and glioma.^[^ ^17^, ^18^, ^19^, ^20^, ^21^, ^22^ ^]^ However, whether the HERV‐K envelope and its cleaved TM subunit, here we collectively refer to these two proteins as K‐TM subunits, could contribute to T cell dysfunction and tumor immune escape is unknown.

AML and PDAC are representatives of hematologic malignancies and solid tumors with no valid response to current immune therapies, such as cell therapy and PD1/PD‐L1 inhibitors. Meanwhile, it has been confirmed that the two tumor types showed high expression of K‐TM, which is associated with tumor prognosis. In this study, we applied AML and PDAC as representatives of hematologic malignancies and solid tumors, respectively, to systematically evaluate the potential role of K‐TM as a representative sample among HERVs accounting for 8% of the human genome in CD8^+^ T cell dysfunction‐mediated tumor immune evasion, and discovered that the K‐TM subunits were potent driver for immune evasion in AML and PDAC. Furthermore, we explore the specific mechanism by which K‐TM regulates T cell function and disclosed that K‐TM bound directly to the ITAM domain of the CD3ε with high affinity via its TMD domain and inhibits phosphorylation of CD3ε. Finally, we discussed the strategy of enhancing the CD8^+^ T cell antitumor function by targeted inhibition of K‐TM, and we found that K‐TM could be targeted to exert enhanced T‐cell antitumor function by either human sera with K‐TM‐reactive antibodies or targeted human recombinant proteins hFL.

As K‐TM subunits are expressed on immune cells, and exhibit immunosuppressive activity like PD‐1, we define the K‐TM subunits as viral immune checkpoints (VICs).

## 2 Results and Discussions

### 2.1 HERV‐K‐Env Proteins with TM Subunit are Aberrantly Expressed in Primary CD8^+^ T Cells and Highly Enriched in Sera of Cancer Patients

**Figure 1:**
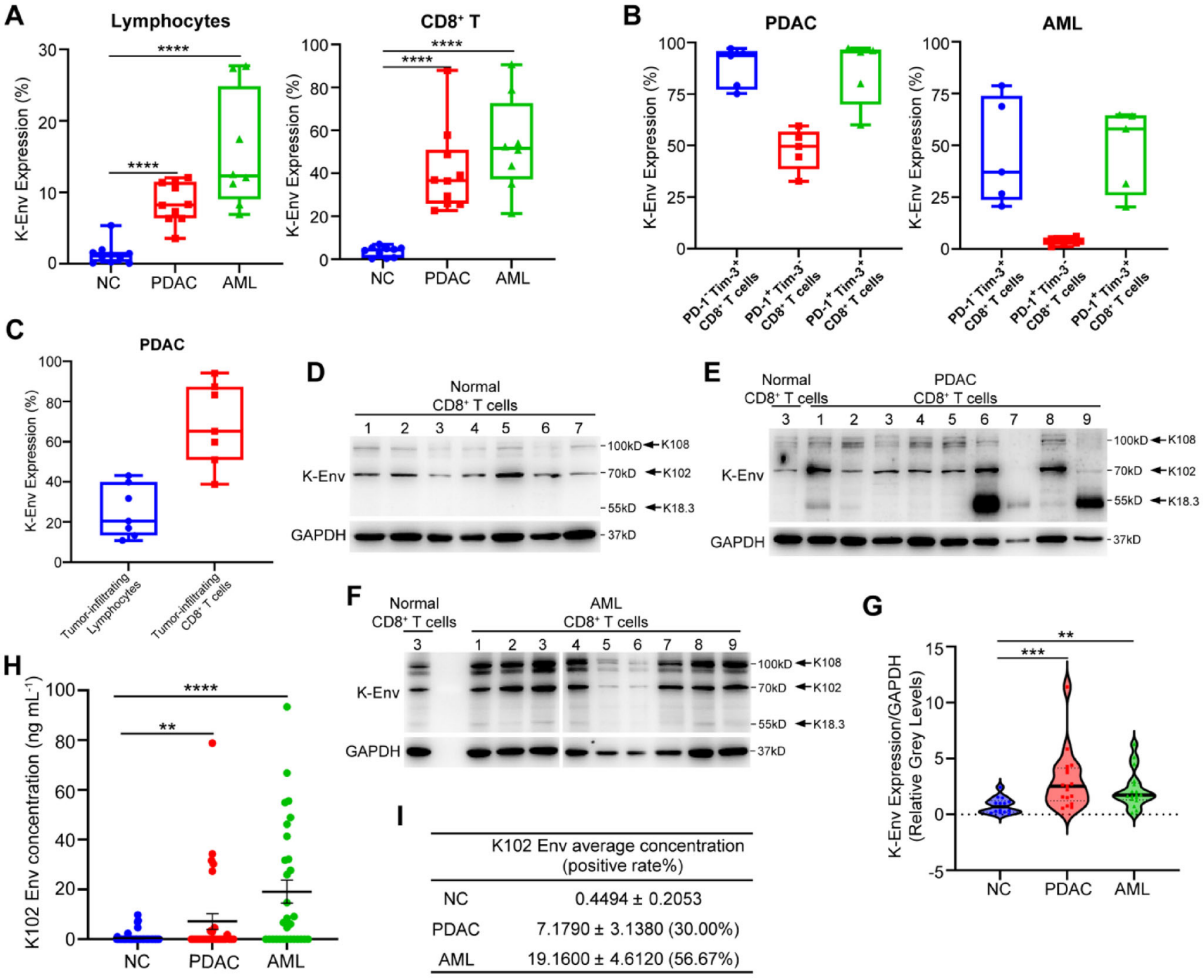
K‐Env is expressed in primary CD8^+^ T cells and enriched in sera of cancer patients. A) Expression of K‐Env in lymphocytes (left) and CD8^+^ T cells (right) from normal subjects (*n* = 10), PDAC (*n* = 10), and AML (*n* = 8) patients was analyzed by Flow Cytometry. B) Expression of K‐Env in PD‐1^‐^ Tim‐3^+^, PD‐1^+^ Tim‐3^‐^, PD‐1^+^ Tim‐3^+^ CD8^+^ T cell subsets from PDAC (*n* = 5, left) and AML (*n* = 5, right) patients was analyzed by Flow Cytometry. C) Expression of K‐Env in tumor‐infiltrating cells (TILs) as well as tumor‐infiltrating CD8^+^ T cells from PDAC patients (*n* = 7) was analyzed by Flow Cytometry. D–F) Western blot analysis of K‐Env in activated CD8^+^ T cells from normal subjects (D, *n* = 7). PDAC patients (E, *n* = 9) and AML (F, *n* = 9). Sample 3 from normal CD8^+^ T cells as the negative control. G) The Image J analysis of the relative quantification of K‐Env protein expression levels. H) ELISA analysis of K102 Env protein levels in the sera of NC (normal subjects) (*n* = 72), AML (*n* = 30), and PDAC (*n* = 30) patients. I) Table enumerates the K102 Env average concentrations and the positive rate of tumor patients compared with the normal. Statistical analyses: Error bars represent mean ± s.e.m., *p* values were calculated by two‐sided student's *t*‐test and **p < 0.01; ***p < 0.001; ****p < 0.0001.

We and other studies have shown that HERV‐K Env and its free TM subunit proteins were aberrantly expressed in tumor tissues of AML and PDAC patients.^[^ ^17^, ^22^ ^]^ Moreover, the K‐TM proteins disappeared in AML patients with complete remission after combination chemotherapy.^[^ ^17^ ^]^ Several HERV TM subunits including K‐TM subunits have potential immunosuppressive properties^[^ ^23^, ^24^, ^25^, ^26^, ^27^ ^]^ and are aberrantly expressed in primary B cells and PBMCs in HIV‐1 infected individuals, but not in normal individuals.^[^ ^28^, ^29^ ^]^ CD8^+^ T cells play critical roles in antitumor immunity, but it is unknown whether K‐TM subunits are expressed in CD8^+^ T cells. Because K‐TM subunits have potential immunosuppressive properties, we hypothesized that K‐TM subunits might play a role in tumor immune escape, if they could interact with CD8^+^ T cells. To test it, we first investigated whether the K‐TM proteins were expressed in primary CD8^+^ T lymphocytes of cancer patients and healthy individuals. Since commercial K‐TM antibody for Flow Cytometry was not available, we applied specific P236186 antibody, which was prepared in our lab and its specificity for K‐Env was confirmed by Enzyme‐Linked Immunosorbnent Assay (ELISA) and flow cytometry (FCM) in our patent (pending ID: 202 411 470 226.8), to detect K‐Env proteins while representing the expression level of K‐TM. K‐Env protein positivity was observed in the majority of lymphocytes and CD8^+^ T cells obtained from peripheral blood of PDAC patients and AML patients as compared with that of healthy individuals. K‐Env expression in CD8^+^ T cells was markedly elevated in PDAC and AML patients and the average frequency of K‐Env^+^ CD8^+^ T cells was 41.07% (22.71%–87.93%) and 53.36% (21.40%–90.72%), while K‐Env^+^ CD8^+^ T double‐positive cells in normal subjects was almost absent and average frequency is 3.53% (0.26%–6.95%) (**Figure** **1**A; and Figure S1A, Supporting Information). We further investigated the correlation between K‐Env expression and CD8^+^ T cell functional states. Elevated K‐Env levels were observed in both PD‐1^‐^ TIM‐3^+^ and PD‐1^+^ TIM‐3^‐^ early exhausted CD8^+^ T cell subsets from cancer patients, as well as PD‐1^+^ TIM‐3^+^ terminally exhausted CD8^+^ T cells. Notably, the highest K‐Env expression was 85.73% (60.00%–97.25%) detected in PD‐1^+^ TIM‐3^+^ terminally exhausted CD8^+^ T cells from PDAC patients (Figure 1B). To extend the above investigation, K‐Env expression was systematically assessed in tumor‐infiltrating lymphocytes (TILs) isolated from PDAC patients. Our results showed K‐Env was aberrantly expressed in both TILs and tumor‐infiltrating CD8^+^ T cells, and the frequency of K‐Env^+^ TILs and K‐Env^+^ tumor‐infiltrating CD8^+^ T cells was 25.24% (10.91%–43.11%) and 68.51% (38.81%–94.06%), respectively (Figure 1C; and Figure S1B, Supporting Information).

Given that effector T cells should be in an activated state when they kill tumor cells, we asked whether K‐Env was further upregulated in activated T cells. Primary CD8^+^ T cells were isolated from normal subjects, PDAC and AML patients, and were activated with CD3/CD28 dynabeads as described in the Experimental Section. We applied specific P233295 antibody, which was prepared in our lab and its specificity for TM subunit of K‐Env was confirmed by TM antigen blocking assay (Figure S2, Supporting Information), to detect K‐Env proteins with western blotting. As expected, K‐Env proteins including K108, K102, or K18.3 subtypes were absent or low in CD8^+^ T cells from healthy individuals, but highly expressed in CD8^+^ T cells from PDAC and AML patients (Figure 1D–G). Notably, the K18.3 Env subtype was specifically expressed in PDAC patients (Figure 1E).

To investigate whether K‐Env proteins were present in the sera of cancer patients, we next measured K102 Env protein levels in the sera of cancer patients and healthy individuals. Unexpectedly, we observed that K102 Env protein levels were markedly increased in sera from 56.67% of AML patients and 30.00% of PDAC patients as compared with that of healthy individuals (Figure 1H,I). The mean values of K102 Env proteins of AML, PDAC, and normal individuals were 19.1600 ± 4.612, 7.1790 ± 3.1380, and 0.4494 ± 0.2053 ng mL^−1^, respectively. In addition, 6.94% of normal individuals (5/72) also showed low levels of K102 Env proteins (2.38–9.80 ng mL^−1^) (Figure 1H,I). These findings indicate that there are at least two K‐Env sources: CD8^+^ T cells expressed‐intracellular K‐Env protein and high levels of extracellular K‐Env protein in sera of cancer patients. Both intracellular and extracellular Env proteins might mediate T cell antitumor function.

### 2.2 K‐TM Subunits are Inversely Correlated with T Cell Antitumor Effect

**Figure 2:**
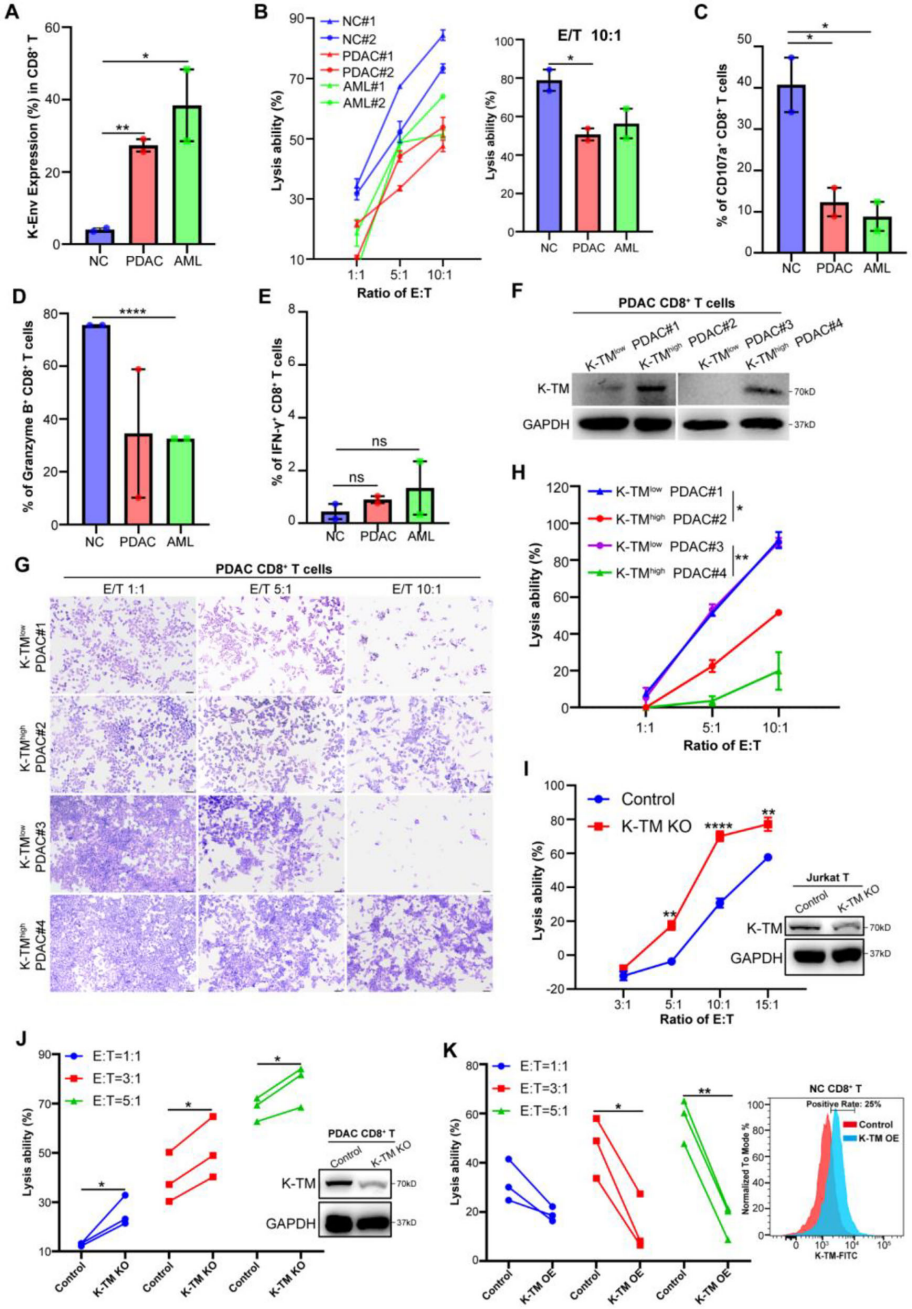
K‐TM expression is inversely correlated with T cell antitumor effect. A) The percentage of K‐Env^+^ CD8^+^ T cells from normal subjects (*n* = 2), PDAC (*n* = 2), and AML (*n* = 2) patients was determined by Flow Cytometry. B) Lysis of MIA‐paca2‐luciferase tumor cell lines (Target cells, T) by CD8^+^ T cells (Effector cells, E) was determined by luciferase‐based cytotoxicity assay at the indicated E:T ratios and data was shown as line graph. Lysis ability of CD8^+^ T cells differed most significantly at a 10:1 target ratio and data was shown separately in a bar chart. C–E) CD8^+^ T cells were co‐cultured with MIA‐paca2 target cells for 5 h, then the frequencies of CD107a‐positive cells C), Granzyme B‐positive cells D), and IFN‐γ‐positive cells E) in each group were detected by Flow Cytometry. F,G) Tumor lysis ability of two pairs of PDAC CD8^+^ T cells with different levels of K‐TM expression F) was detected by crystal violet assay after 48 h‐incubation with MIA‐paca2 cells, and representative images were shown in G). Scale bars: 40 µm. H) Lysis ability of CD8^+^ T cells calculated by viable cells quantified with crystal violet staining. I) The lysis ability of Jurkat^Control^ and Jurkat^K‐TM KO^ cells (Effector cells, E) in MIA‐paca2 tumor cells (Target cells, T) at the indicated E:T ratios. Control: nontargeting cells. K‐TM KO: K‐TM knockout. J) The lysis ability of CD8^+^ T^Control^ cells and CD8^+^ T^K‐TM KO^ cells (Effector cells, E) from PDAC patients (*n* = 3) in MIA‐paca2 tumor cells (Target cells, T) at the indicated E:T ratios. Control: nontargeting cells. K‐TM KO: K‐TM knockout. K) The lysis ability of CD8^+^ T^Control^ and CD8^+^ T^K‐TM OE^ cells (Effector cells, E) from normal subjects (*n* = 3) in MIA‐paca2 tumor cells (Target cells, T) at indicated E:T ratios. K‐TM OE: K‐TM overexpression. Statistical analyses: Data were shown as mean ± s.e.m. with triple repetitions, statistical significance was determined by Two‐Way ANOVA or two‐sided student's *t*‐test, *p < 0.05; **p < 0.01; ****p < 0.0001; and ns *p* > 0.05.

Given that T cells in cancer patients are frequently incapable of killing tumor cells, to test whether K‐TM subunit was involved in the loss of T cell antitumor effect, we next investigated whether there was a correlation between K‐TM level and tumor lysing ability of CD8^+^ T cells. We first compared T cell antitumor abilities from normal subjects with low K‐TM protein and patient samples, including PDAC and AML with high K‐TM protein (**Figure** **2**A). Differed T cells were co‐cultured with MIA‐paca2‐luciferase tumor cells to explore their cytolytic capacity applying Luciferase Assay, we observed that CD8^+^ T cells with low K‐TM from the healthy subject exhibited potent tumor‐killing activity in a dose‐dependent manner and lysed nearly most tumor cells at the 10:1 ratio of E/T (effector T cell/target tumor cell). In contrast, CD8^+^ T cells with high K‐TM from PDAC and AML patients showed weak antitumor activity (Figure 2B). We also measured the CD107a degranulation and cytokines release to further evaluate T cell function. Consistent with the above results, CD8^+^ T cells with low K‐TM expression from normal subjects demonstrated significantly increased CD107a degranulation (Figure 2C) and more Granzyme B cytokine release, but not IFN‐γ (Figure 2D,E). To confirm the result, we further compared CD8^+^ T cells with high or low K‐TM from PDAC applying Crystal Violet Staining Assay. Similar results were observed in primary CD8^+^ T cells of PDAC (Figure 2F–H).

To better explore the specific relationship between K‐TM expression and T cell antitumor abilities, we induced K‐TM silence both on human Jurkat T cells and primary CD8^+^ T cells using CRISPR‐Cas9 technology. Native Jurkat T cells are under anergy and have no antitumor action, but their tumor‐killing ability could be reversed by PMA/ionomycin (P/I) cocktail stimulation. We first established K‐TM knockout (KO) Jurkat T cell lines (Jurkat‐K‐TM^KO^), and compared its tumor lysis ability with the control Jurkat T cells. As expected, Jurkat‐K‐TM^KO^ T cells elicited stronger cytotoxicity against tumor cells compared to the control (Figure 2I). Using Electroporation and CRISPR‐Cas9 technology, we simultaneously constructed K‐TM^KO^ CD8^+^ T cells from PDAC patients. We observed similar results in primary CD8^+^ T cells, K‐TM^KO^ CD8^+^ T cells eliminated Mia‐paca2‐luciferase tumor cells significantly better than control (Figure 2J). In addition, K‐TM was transiently overexpressed in CD8^+^ T cells prior to activation and then detected its effect on T cell cytotoxic activity, quantitative analysis revealed significant attenuation of target cell lysis capacity in K‐TM‐overexpressing T cells compared to vector control (Figure 2K). The above results suggest that the K‐TM could be a negative regulator of antitumor responses.

### 2.3 K‐TM Subunits Induce CD8^+^ T Cell Anergy and Impair T Cell Antitumor Function

T cell anergy is regarded as immune tolerance in which the lymphocyte is functionally inactivated, but remains alive for an extended period in a hyporesponsive state. We next assessed whether K‐TM affected the antitumor function of T cells. We first established K‐TM‐high expressing Jurkat T cells and assessed whether K‐TM expression affects the growth of Jurkat T cells using MTT assay. We observed that K‐TM expression significantly inhibited the growth of Jurkat T cells (Figure S3A,B, Supporting Information). However, FCM results showed that K‐TM‐expressing Jurkat T cells are not apoptotic or death (Figure S3C,D, Supporting Information). These results suggest that K‐TM protein inhibits T cell activation and proliferation, but does not affect cell viability. We next investigated whether K‐TM expression affected the tumor lysis ability of P/I‐activated Jurkat T cells using coculture of Jurkat T cells with PDAC MIA‐paca2 cells (E/T ratios ranging from 2:1 to 5:1). Jurkat T cells overexpressed with K‐TM or empty vector were cocultured with MIA‐paca2 cells with GFP tag in the presence of P/I cocktail and tumor‐killing activities were examined with fluorescence microscope. As expected, native Jurkat T cells had no antitumor activity, and P/I cocktail‐stimulated Jurkat T cells exhibited potent tumor lysing activity against MIA‐paca2 (Figure S3E, Supporting Information). In contrast, the tumor killing activity of TM‐high expressing Jurkat T cells decreased by 40% of control Jurkat T cells at the 5:1 E/T ratio under stimulation of P/I (Figure S3F, Supporting Information). These findings indicate that K‐TM expression inhibits the proliferation of T cells and impairs its antitumor function, but do not affect T cell viability.

**Figure 3:**
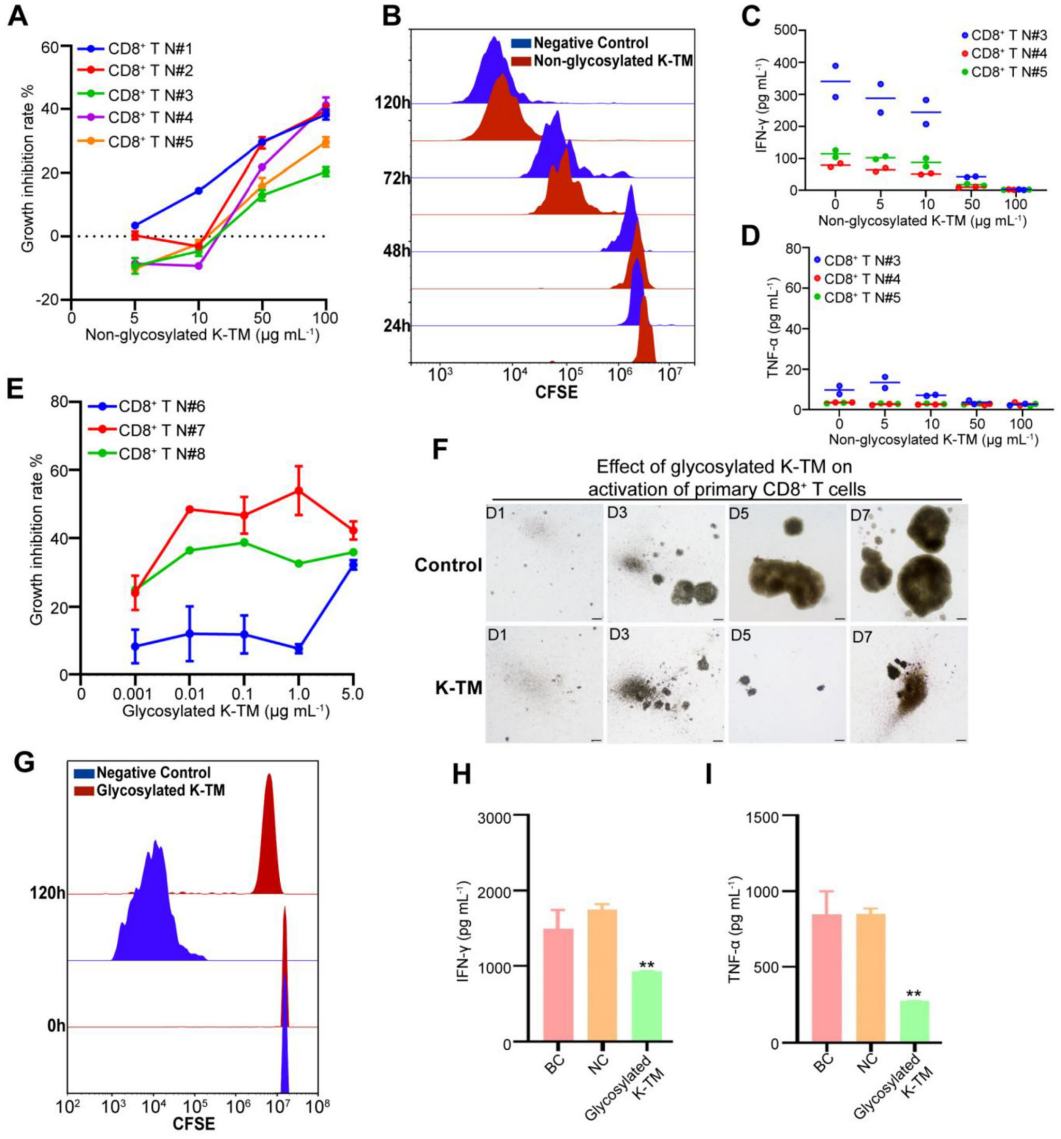
K‐TM protein blocks CD8^+^ T cell activation and cytokine INF‐γ release. A) T cell growth inhibition assay showing inhibition of nonglycosylated K‐TM protein on T cell proliferation in a dose‐dependent manner (*n* = 5). B) CFSE assay showing inhibition of nonglycosylated K‐TM protein (10 µg mL^−1^) on T cell proliferation. C,D) Detection of cytokine effector IFN‐γ C) and TNF‐α D) production in CD8^+^ T cells treated with nonglycosylated K‐TM protein. E) T cell growth inhibition assay showing inhibition of glycosylated K‐TM protein on T cell proliferation in a dose‐dependent manner (*n* = 3). F) Representative figures showing inhibition of glycosylated K‐TM protein on T cell proliferation. Scale bars: 100 µm. G) CFSE assay showing that treatment with glycosylated K‐TM protein (1 ng mL^−1^) for 5 days significantly inhibited T cell proliferation. H,I) Detection of cytokine effector IFN‐γ H) and TNF‐α I) production in CD8^+^ T cells treated with glycosylated K‐TM. Statistical analyses: Error bars represent mean ± s.e.m. with three independent experiments, and the statistical significance was determined by two‐sided student's *t*‐test, **p < 0.01.

To confirm the above observations, we next investigated whether the K‐TM subunit protein inhibit primary T cell proliferation and cytokine release. Since both nonglycosylated and glycosylated K‐TM proteins are present in cancer patients, we first evaluated the effects of nonglycosylated K‐TM protein. Primary CD8^+^ T cells isolated from healthy donors were treated with CD3/CD28 beads in the presence of purified recombinant nonglycosylated K‐TM protein from the prokaryotic‐expressed system for 7 days. We found that the nonglycosylated K‐TM proteins could inhibit the proliferation of CD8^+^ T cells in a dose‐dependent manner (**Figure** **3**A). We also applied CFSE assay to further clarify the effect of K‐TM on T cell activation and expansion. CD8^+^ T cells were freshly isolated and maintained in nonactivated state through the CD3/CD28 deprivation, then preincubated with 10 µg mL^−1^ nonglycosylated K‐TM protein for 12 h followed with CFSE labeling. CD8^+^ T cells treated with K‐TM protein exhibited a certain degree of inhibition on the proliferation ability (Figure 3B). We also measured T cell activation markers CD25, CD69, and T cell exhaustion marker PD‐1, the results showed that nonglycosylated K‐TM protein treatment could slightly inhibit activation molecules and upregulate the exhaustion molecule (Figure S3G, Supporting Information). Moreover, the nonglycosylated K‐TM treatment significantly reduced cytokine effector IFN‐γ in CD8^+^ T cells (Figure 3C), but did not affect cytokine effector TNF‐α (Figure 3D).

We then evaluated the effects of glycosylated K‐TM protein on the activation and proliferation of primary CD8^+^ T cells. Primary CD8^+^ T cells were treated with CD3/CD28 beads in the presence of purified recombinant glycosylated K‐TM protein from Hek293T cells for 7 days. As presented in Figure 3E, glycosylated K‐TM protein blocked the CD3/CD28 beads‐mediated activation and proliferation of CD8^+^ T cells in a dose‐dependent manner. The concentration of glycosylated K‐TM protein that functioned was much lower than that of nonglycosylated K‐TM protein. Furthermore, while monitoring cell proliferation with a microscope, we found that 1 ng mL^−1^ glycosylated K‐TM protein could markedly block the proliferation of primary CD8^+^ T cells (Figure 3F). Subsequent CFSE assay further support the above results (Figure 3G). Consistently, glycosylated K‐TM protein notably induced decrease in cytokine release of IFN‐γ and TNF‐α (Figure 3H,I). These data suggest that the glycosylation of K‐TM protein is important for its impairment of T cell function.

Collectively, our results show that the K‐TM subunit induces CD8^+^ T cell anergy by blocking activation and cytokine production of CD8^+^ T cells and impairs its antitumor effect.

### 2.4 K‐TM Subunit Elicits Potent Tumor Immune Escape in Mouse Model

**Figure 4:**
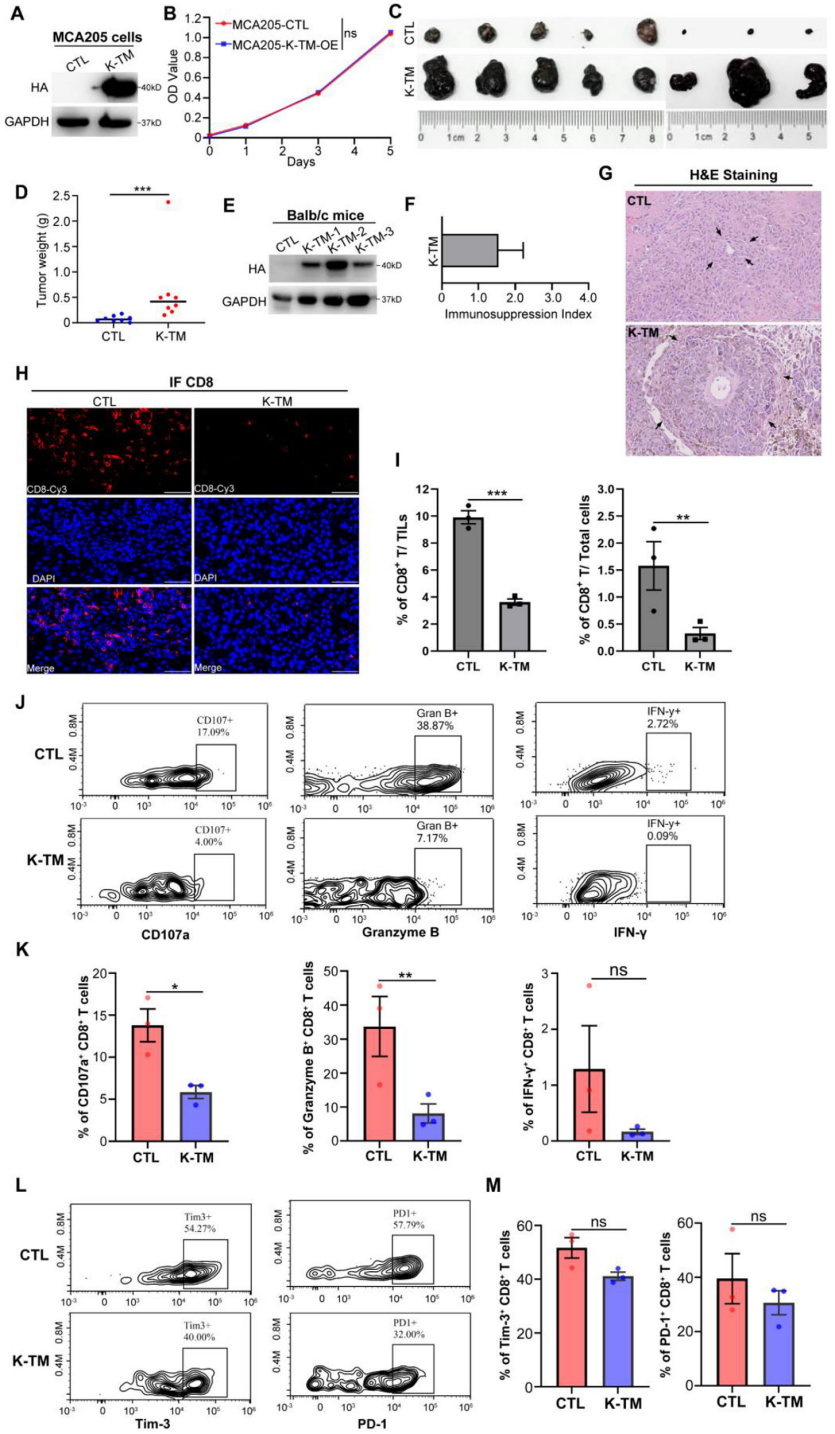
K‐TM elicits potent tumor immune escape in mouse model. A) Western blot analysis of K‐TM protein expression in MCA205 tumor cells confirmed by HA‐tag. B) Comparison of MCA205 cell proliferation activity among K‐TM or empty vector, assessed by MTT with triple repetitions. C) Gross appearance of MCA205 tumors over‐expressed with K‐TM or vector control after xenografting into Balb/c mice at indicated time (*n* = 8). D) Average weight of MCA205 xenografts in Balb/c mice at day 20. E) Western blot analysis of K‐TM protein level in xenograft tumors. F) Immunosuppression index of K‐TM overexpressed group compared with control group. G,H) Representative images of H&E G) and CD8^+^ T cells staining H) in xenografts. Scale bars: 50 µm. I) Quantification of the percentage of CD8^+^ T cell infiltration in TILs (left) and total cells (right) distributed in MCA205 xenografts by FCM (*n* = 3). J–M) FCM analysis detecting the CD107a^+^ CD8^+^ T cell infiltration level (J and K, left), intracellular cytokine of Granzyme B (J and K, medium), and IFN‐γ (J and K, right) on CD8^+^ gated cells, apoptosis‐related molecule Tim‐3 (L and M, left), and exhaustion‐related molecule PD‐1 (L and M, right) on CD8^+^ gated cells (*n* = 3). Statistical analyses: Error bars represent means ± s.e.m., statistical significance was determined by a two‐tailed *t*‐test, *p < 0.05; **p < 0.01; ***p < 0.001; and ns *p* > 0.05.

Given that the immunosuppressive (IS) function is carried by the transmembrane (TM) subunit, both K‐Env and free K‐TM proteins have potential immunosuppressive (IS) activity.^[^ ^23^ ^]^ Notably, we have demonstrated the presence of high levels of K‐Env proteins in the sera of cancer patients as mentioned above. We next investigated whether the K‐TM subunit enables cancer cells to escape immune surveillance in immunocompetent mice using an in vivo tumor‐rejection assay.^[^ ^30^ ^]^ MCA205 cells derived from C57BL mice were transfected with inducible lentivirus expression vectors for the K‐TM subunit and then injected subcutaneously into allogeneic Balb/c mice, and cells expressed with empty expression vector were used as a negative control (**Figure** **4**A). In vitro cell proliferation assay confirmed that K‐TM overexpression elicited no increased proliferation in MCA205 cells (Figure 4B). We observed that tumors with K‐TM^high^ expression in Balb/c mice (*n* = 8) grew much faster (Figure 4C–E), at day 20, the average tumor weight was 0.61 ± 0.25 g. By contrast, tumors without K‐TM expression grew slowly, the average tumor weight was 0.07 ± 0.02 g (Figure 4C,D). We further quantified immunosuppressive (IS) activity by an index based on tumor size: (A_K‐TM_ – A_CTL_)/A_CTL_, where A_K‐TM_ and A_CTL_ were the mean areas at the peak of growth of tumors from BALB/c mice injected with K‐TM‐expressing or control cells, respectively. A positive index indicates that K‐TM expression facilitates tumor growth. We found that the immunosuppression index of K‐TM was 1.54 (Figure 4F), suggesting that the free K‐TM subunit exhibits potent IS activity. H&E staining showed that more cellular atypia with a higher mitotic rate, more clearly formed and larger cancer nests were found in the K‐TM subunit expressed MCA205 xenografts, as compared to the control group (Figure 4G).

Given that both PDAC and AML are considered to be immunologically “cold” tumors, their microenvironment is characterized by a lack of cytotoxic T cells. To reveal the possible mechanism by which T cells were involved in K‐TM‐mediated immunosuppression, the infiltration level estimation and functional analysis of tumor‐responsive CD8^+^ TILs were conducted through IHC and FCM methods. We observed that CD8^+^ T was abundantly in control tumor tissues, but markedly reduced in K‐TM‐expressing tumor tissues (Figure 4H,I), accompanied by an upregulation in CD107a degranulation and Granzyme B cytokines release, but not IFN‐γ (Figure 4J,K), supporting our in vitro findings that K‐TM protein blocked the activation and proliferation of CD8^+^ T cells. Moreover, we examined the apoptosis‐related molecule Tim‐3 and the exhaustion‐related molecule PD‐1 gating in CD8^+^ T cells, no difference was found between the K‐TM‐expressing tumor group and the control tumor group, indicating K‐TM subunit function not by inducing apoptosis and exhaustion of CD8^+^ T cells (Figure 4L,M). Collectively, the results indicate that the K‐TM subunit expression in MCA205 cells may contribute to the development of the immunologically “cold” microenvironment.

Taking the results together, our findings demonstrate that the K‐TM subunit enables MCA205 tumor cells to escape immune surveillance in allogeneic and immune‐competent Balb/c mice.

### 2.5 K‐TM Subunit Binds to the ITAM of CD3ε Coreceptor via Its TMD and Inhibits Phosphorylation of CD3ε

**Figure 5:**
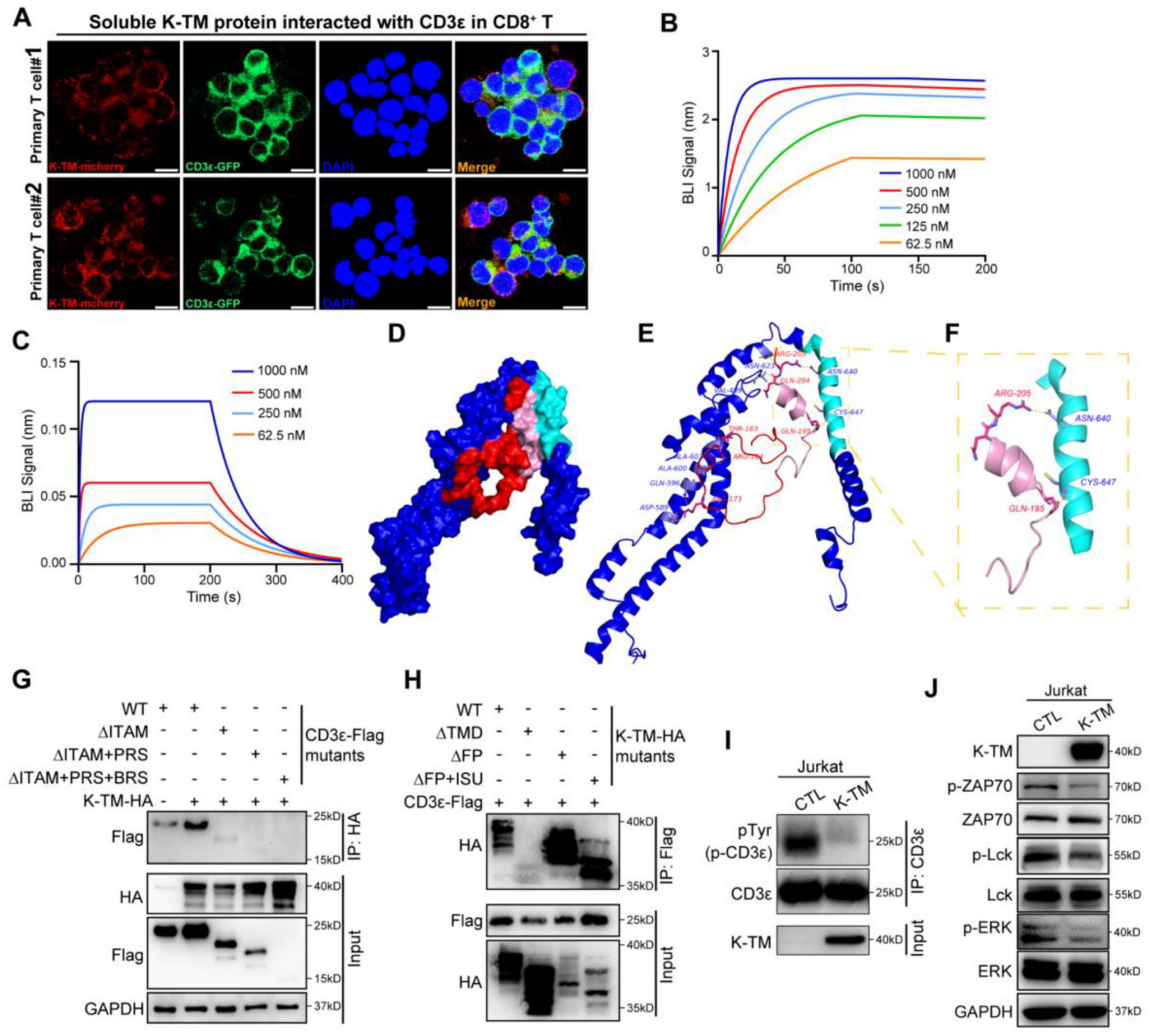
K‐TM binds to the ITAM of CD3ε coreceptor via its TMD. A) Colocalization of soluble K‐TM‐mCherry (red) interacted with CD3ε‐GFP (green) on CD8^+^ T cell membrane (*n* = 2). Scale bars: 10 µm. B,C) BLI assay showing binding affinity of glycosylated K‐TM protein to glycosylated CD3ε protein B), nonglycosylated K‐TM protein to nonglycosylated CD3ε protein C) in a dose‐dependent manner. D) K‐TM protein (blue) binds tightly to CD3ε (red) in the Docking Model. TMD domain of K‐TM and ITAM domain of CD3ε are colored as light‐blue, and pink, respectively. E) Interaction of K‐TM (blue) in complex with CD3ε (red) in the Docking Model. The complex forms eight hydrogen bonds (shown as yellow dots) involving eight pairs of highlighted amino acids, while part of K‐TM is labeled as blue and CD3ε labeled as red. F) Interaction of K‐TM TMD domain (light blue) in complex with CD3ε ITAM domain (pink) in the Docking Model. The complex forms two hydrogen bonds with the closest interaction distance (shown as yellow dots). Two pairs of amino acids from K‐TM and CD3ε were highlighted and labeled as blue and red. G) Binding site mapping of K‐TM‐HA to CD3ε protein using Flag‐tagged CD3ε (CD3ε‐Flag) protein mutants and co‐IP assay. WT: CD3ε wild‐type protein. H) Binding site mapping of CD3ε‐Flag to K‐TM protein using HA‐tagged K‐TM (K‐TM‐HA) protein mutants and co‐IP assay. WT: K‐TM wild‐type protein. I) Comparison of phosphorylation level of endogenous CD3ε changes in Jurkat T cells by K‐TM overexpression. J) Immunoblot of ZAP70/Lck/ERK phosphorylation changes in Jurkat T cells by K‐TM.

CD3ɛ is crucial for T cell development not shared by the CD3γ, CD3δ, or ζ‐family proteins, and CD3ɛ‐knockout mice exhibit T cell deficiency.^[^ ^31^ ^]^ Therefore, we next focused on whether the CD3ε is the potential target of the K‐TM subunit in T cells. We applied K‐TM overexpressing Jurkat T cells for co‐IP assay and observed that the K‐TM protein interacted with endogenous CD3ε in Jurkat T cells (Figure S4A, Supporting Information). To further confirm this interaction, expression vector pcDNA3.1 encoding K‐TM protein with HA‐tag and CD3ε protein with Flag‐tag, respectively, were generated and cotransfected Hek293T cells, then collected for co‐IP assay. Consistently, K‐TM protein was pulled down by CD3ε, and CD3ε was reversely pulled down by K‐TM (Figure S4B,C, Supporting Information). To reveal the distribution of K‐TM protein in T cells, Jurkat T cells were expressed with K‐TM‐GFP fusion protein to investigate the localization of K‐TM and endogenous CD3ε under confocal microscope. We found that K‐TM was predominantly colocalized with CD3ε on the cell membrane of Jurkat T cells (Figure S4D, Supporting Information). Given the presence of high levels of K‐TM soluble protein in plasma, we further investigated whether the extracellular domain of K‐TM could directly interact with T cells. Jurkat T cells and nonactivated CD8^+^ T cells were incubated with mCherry‐tagged K‐TM protein, then stained with fluorescence‐labeled CD3ε antibody, IF assay revealed that the soluble K‐TM protein could directly interact with the CD3ε on T cell membrane (**Figure** **5**A; and Figure S4E, Supporting Information). Similar to HIV‐1 gp41 fusion peptide (FP), which inserts into the T cell membrane and blocks TCR/CD3 interaction,^[^ ^32^ ^]^ we hypothesized K‐TM's conserved FP domain mediates the same process. IF assay confirmed that soluble FP protein colocalized with CD3ε on both Jurkat and primary T cells, indicating that K‐TM inserts via its FP domain to disrupt TCR/CD3 signaling (Figure S4F,G, Supporting Information). To verify whether CD3ε is the direct target of K‐TM protein, we applied Bio‐Layer Interferometry (BLI) to determine the binding affinity between K‐TM protein and CD3ε protein. Recombinant protein were purified from eukaryotic or prokaryotic‐expressed system, respectively (Figure S5A,B, Supporting Information). We observed that the glycosylated K‐TM protein bound to CD3ε with a *K*_D_ of 1.83 nm (Figure 5B), while the nonglycosylated K‐TM protein bound to CD3ε with a *K*_D_ of 67.7 nm (Figure 5C), the affinity of nonglycosylated K‐TM protein with CD3ε decreased by 36.99‐fold of glycosylated K‐TM protein. These results show that K‐TM protein directly binds to the CD3ε protein with a nanomolar affinity and glycosylation is critical for functional K‐TM protein.

To further confirm the above observations, we built a docking model of CD3ε with K‐TM. The homology model of CD3ε protein in the range of 153–207 aa was built by Swiss‐Model, and the homologous modeling of K‐TM ranging from 466 to 699 aa by AlphaFold2, since so far there is no X‐ray structure for the predicted binding site. Consistently, we observed that CD3ε and K‐TM exhibited a tight binding score of ‐401.22 kcal mol^−1^, while hydrogen bonds formed the most hydrophobic interaction network (Figure 5D–F). The best binding pocket sites for CD3ε‐K‐TM, such as GLN195‐CYS647, and ARG205‐ASN640, were located in the CD3ε 185–205 domain and K‐TM 633–653 residues (Figure 5E,F). Meanwhile, in order to investigate the mechanism of CD3ε binding to K‐TM protein, we applied Gromacs2020 software to analyze molecular dynamics of the protein–protein complexes. As shown in Figure S5C (Supporting Information), RMSD of the complexes was less than 1.1 nm, the complexes basically reached a dynamic equilibrium at around 60 ns, indicating that the proteins were well‐matched and could form stable complexes. RMSF plots (Figure S5D,E, Supporting Information) showed large conformational changes in some amino acids, mainly due to that the amino acids in this part were located in the hinge region of the protein, more flexible and the conformation was more susceptible to change during the simulation process. We further constructed a series of HA‐tagged K‐TM deletion mutants and Flag‐tagged CD3ε deletion mutants, and determined the binding ability of HA‐tagged K‐TM deletion mutants to CD3ε deletion mutants in Hek293T cells. We found that the K‐TM TMD domain strongly interacted with the ITAM domain of CD3ε, which was the only phosphorylation site of CD3ε, but not with other domains: BRS and PRS (Figure 5G,H). CD3‐TCR phosphorylation is the first signaling event in T cells to elicit adaptive immunity against invading pathogens and tumor cells. We applied Jurkat cells, a widely adopted model for studying T cell signaling with fewer experimental variations compared to primary T cells,^[^ ^33^, ^34^ ^]^ to investigate K‐TM function on phosphorylation levels of CD3ε and the subsequent TCR‐signaling. We found that the phosphorylation level of CD3ε decreased upon the expression of K‐TM in Jurkat T cells (Figure 5I). Subsequently, we examined the phosphorylation of ZAP70, Lck and ERK (TCR‐CD3 downstream signaling molecule) in Jurkat T cells after K‐TM expression, and indicated that K‐TM could attenuate the phosphorylation of ZAP70, Lck, and ERK (Figure 5J). These results indicate that CD3ε is a critical target of the K‐TM protein that directly binds the CD3ε ITAM domain via K‐TM TMD and the K‐TM might disable the TCR signaling by inhibiting CD3ε phosphorylation (Figure S5F, Supporting Information).

### 2.6 K‐TM Proteins are Highly Conserved in the Human Genome

It is unknown about the exact members of functional K‐TM proteins in the human genome. Up to now, only a functional Env protein from HERV‐K108 provirus has been described,^[^ ^35^ ^]^ although previous studies revealed a probable total of about 550 HERV‐K proviruses in the human genome.^[^ ^36^ ^]^ Thus, we next searched all potential open reading frames (ORFs) encoding K‐TM subunits in the human genome and then characterized these K‐TM protein sequences, especially three putative immunosuppressive domains: FP, ISU, and TMD (Figure S6A, Supporting Information). We identified a total of 67 intact ORFs for K‐TM protein, which are distributed in chr 1, chr 2, chr 3, chr 5, chr 6, chr 7, chr 8, chr 12, chr 13, chr 16, chr 19, chr 22, and chr X (Table S1, Supporting Information).

Unlike HERV‐K Env SU subunits, which are various in the HERV‐K family, alignment analysis showed that amino acid sequences of K‐TM proteins were highly similar with more than 98% identity at the amino acid level, suggesting that K‐TM proteins are conserved in the human genome. We found that all K‐TMs contain three putative immunosuppressive domains of FP, ISU, and TMD (Figure S6, Supporting Information). While, notably, the entire FP domain FIFTLIAVIMGLIAVTATAAV, the ISU domain LANQINDLRQTVIWMGD, and the TMD domain IGSTTIINLILILVCLFCLLL were highly conserved throughout all K‐TM proteins with more than 70%–98% identity. In rare cases, the mutant sequences LANQISDLRQTVIWMRD in the ISU domain were found (Figure S6B, Supporting Information), indicating the high conservation of these domains in all HERV‐K proviruses in the human genome. These data suggest that K‐TM proteins might be critical for triggering immune evasion in tumors.

### 2.7 Targeting of K‐TM Improves CD8^+^ T Cell Antitumor Efficacy In Vitro and In Vivo

**Figure 6:**
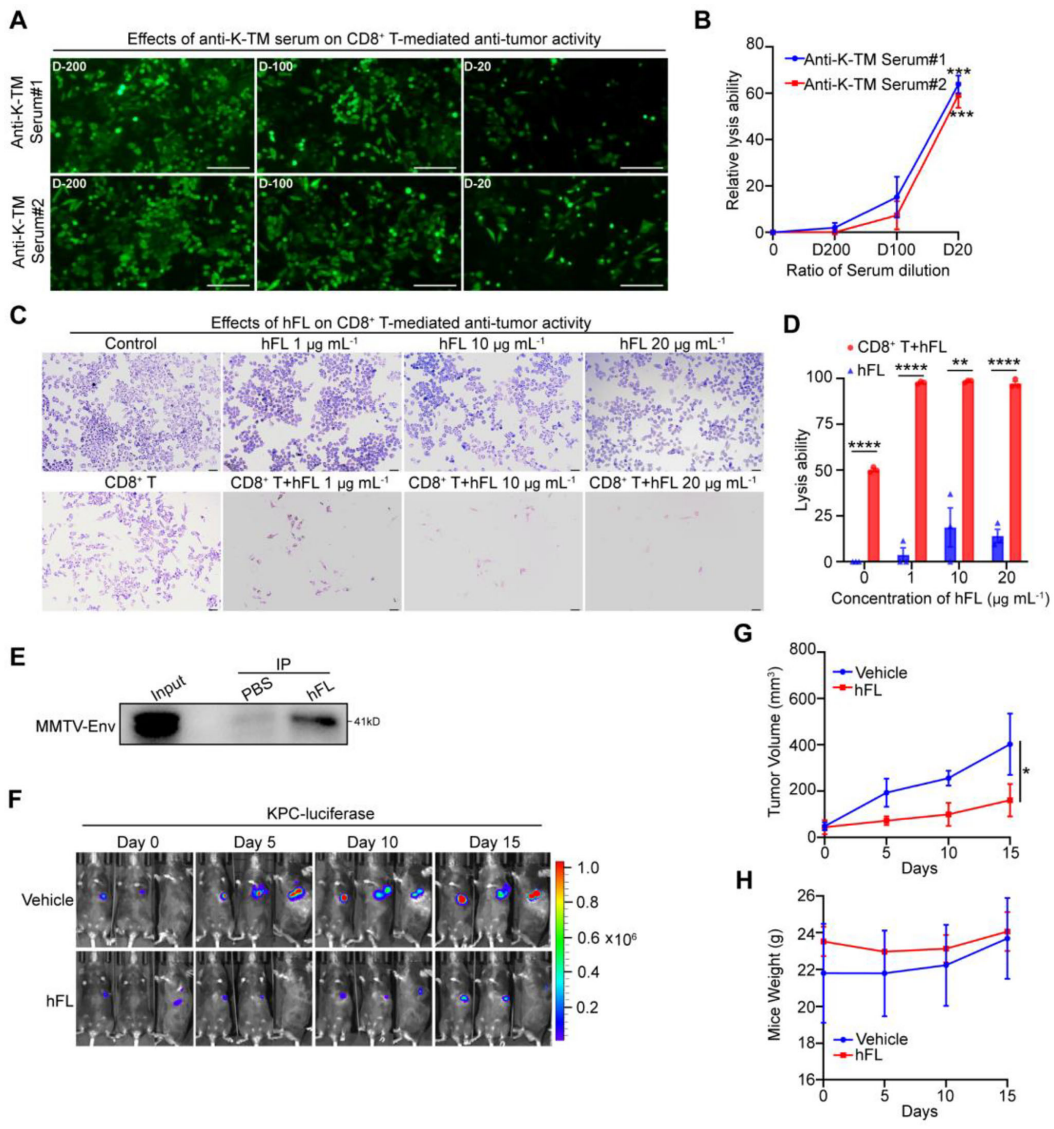
Targeting K‐TM improves CD8^+^ T cell antitumor efficacy in vitro and in vivo. A) Effects of K‐TM‐reactive antibodies on the lysis ability of K‐TM‐high CD8^+^ T cells. D‐200: 1/200 dilution, D‐100: 1/100 dilution, and D‐20: 1/20 dilution. B) Quantification of CD8^+^ T cell lysis ability after treatment with indicated dilution ratios of K‐TM antibody positive serum. Statistical significance of the D20 group and D200 group. C) hFL enhances killing ability of CD8^+^ T cells. CD8^+^ T cells were and pretreated with hFL and then incubated with MIA‐paca2 cells for 48 h at 5:1 (E:T). Surviving cells were stained with crystal violet. D) Lysis ability of CD8^+^ T cells was quantified after treatment with hFL. E) Co‐IP assay of hfL coupled with HA‐tag to endogenous MMTV protein in KPC cells using HA‐tagged beads. PBS: negative control. F) Bioluminescence imaging of KPC xenograft C57BL mice treated with vehicle or 5 mg kg^−1^ hFL (*n* = 3). G,H) Tumor volume of KPC xenografts G) and mouse weight H) after treatment with vehicle or 5 mg kg^−1^ hFL at indicated time. Statistical analyses: Error bars represent mean ± s.e.m. with three independent experiments, and the statistical significance was determined by the two‐tailed *t*‐test. **p < 0.01; ***p < 0.001; ****p < 0.0001.

Finally, we asked whether targeting K‐TM could reverse CD8^+^ T cell anergy, and restore T cell‐mediated tumor cell killing. To this end, human sera with K‐TM‐reactive antibodies were collected from immune‐hyperactive systemic lupus erythematosus (SLE) patients and antibody potency was determined by ELISA assay with specific K‐TM antigen. As shown in the results, sera from SLE patients contained high titers of K‐TM‐reactive antibodies compared to normal subjects (Figure S7A, Supporting Information). We further treated K‐TM‐high CD8^+^ T cells isolated from PDAC patients using the above human sera with K‐TM‐reactive antibodies for 24 h and then investigated tumor lysis ability. We found that the serum with K‐TM‐reactive antibodies exhibited ADCC against PDAC MIA‐paca2 in a dose‐dependent manner, and over 60% of MIA‐paca2 cells were lysed after serum treatment at 1:20 dilution (**Figure** **6**A,B), suggesting that antibody‐mediated targeting of K‐TM markedly enhances CD8^+^ T cell antitumor function.

To validate these observations, we next investigated whether targeting K‐TM protein with a mannose‐binding lectin hFL could affect the tumor lysis ability of CD8^+^ T cells via coculturing CD8^+^ T cells with PDAC MIA‐paca2 cells (E:T = 5:1). hFL lectin bounds to K‐TM protein directly with a high affinity of 49.5 nM (Figure S7B,C, Supporting Information. Furthermore, we observed that hFL (green) was specifically co‐localized with K‐TM‐mCherry (red) in K‐TM‐overexpressed Hek293T cells after treatment with hFL lectin (Figure S7D, Supporting Information). Moreover, hFL lectin treatment could markedly improve the CD8^+^ T cell tumor lysing activity against Mia‐paca2 cells in a dose dose‐dependent manner (Figure 6C,D), while single hFL showed no obvious cytotoxicity against cancer cells at the tested concentrations.

To further confirm whether targeting K‐TM by the hFL lectin inhibits the growth of PDAC cells in vivo, murine KPC cells harboring mutant KRAS_G12D_ and TP53_R172H_ and active MMTV‐Env (Figure 6E), which was closely related to HERV‐K,^[^ ^37^ ^]^ were injected subcutaneously into the immunocompetent C57BL mice. After the xenografted tumor signals could be detected, mice were randomized to two groups to receive either vehicle or hFL lectin treatment via i.p administration. Consistent with the above results, hFL‐mediated target TM also potently inhibited the growth of PDAC in mice models. As presented in Figure 6F,G, a dramatic reduction in tumor signal and tumor volume was observed with the treatment of hFL lectin at 5.0 mg kg^−1^ dose compared with the group treated with the vehicle. On day 15, the TGI (tumor growth inhibition) of the hFL‐treated group was up to 80% of the control (Figure 6G). hFL lectin at 5.0 mg kg^−1^ dose was also well tolerated by the mice with no significant body weight loss observed (Figure 6H).

Taken together, these data indicate targeting K‐TM reverses CD8^+^ T cell anergy, and restores T cell‐mediated tumor cell killing in vitro and in vivo.

## 3 Conclusion

Here we reveal that the glycosylated K‐TM subunit in T cells and sera of cancer patients is a crucial viral immune checkpoint (VIC) that induces CD8^+^ T cell anergy via directly targeting CD3ε and disabling TCR signaling and enabling cancer cells to escape immunosurveillance. Importantly, targeting K‐TM subunits can reverse CD8^+^ T cell anergy, restore T cell‐mediated tumor cell killing, and regress tumors in animal models.

Increasing studies suggest that HERVs may play critical roles in human cancer,^[^ ^38^, ^39^, ^40^ ^]^ but no solid evidence supports this hypothesis due to the lack of defined viral proteins linked to specific phenotypes of cancers. In this study, we for the first time demonstrate that CD8^+^ T cells from cancer patients express endogenous glycosylated K‐TM subunits with immunosuppressive domains. Moreover, high levels of glycosylated K‐TM subunits are present in sera of AML and PDAC patients. In contrast, glycosylated K‐TM subunits are low or absent in CD8^+^ T cells and sera from healthy individuals. In humans, HERVs are normally silenced and are considered as “junk DNA.” However, certain specific subsets of HERVs like syncytin‐1, syncytin‐2, and HERV‐K are known to have physiological immunosuppression during placental development and for the immune protection of the fetus.^[^ ^26^, ^27^, ^41^ ^]^ Thus, further research is needed on how this pathological elevation of glycosylated K‐TM subunits occurs in cancer patients.

We demonstrate that the K‐TM proteins induce T cell anergy. K‐TM‐low CD8^+^ T cells exhibit potent tumor lysis ability whereas K‐TM‐high T cells are unable to kill tumor cells. The K‐TM subunit protein blocks activation of CD8^+^ T cells, reduces cytokine effector IFN‐γ, and impairs T cell antitumor function, resulting in CD8^+^ T cell anergy. Notably, the K‐TM protein elicits potent tumor immune escape in immunocompetent mouse models. In addition, K‐TM reduces the number of infiltrating CD8^+^ T cells in tumors, leading to “cold” immunosuppressive microenvironment. An unanticipated finding is that K‐TM targets directly to CD3ε of the TCR complex with nanomolar affinity, and inhibits TCR phosphorylation. The TCR phosphorylation is the first signaling event in T cells to elicit adaptive immunity against tumor cells.^[^ ^42^, ^43^, ^44^ ^]^ These findings indicate that the K‐TM triggers CD8^+^ T cell anergy and tumor immune evasion by targeting the coreceptor CD3ε and blocking TCR signaling. Although it is difficult to pinpoint the exact amount of K‐TM subunits contributing to immune evasion in cancer patients, we can speculate that both the number and the amount of total HERV TM subunits might be astonishing based on the fact that HERVs comprising 8% of the human genome. We demonstrate that HERV K102‐Env and K108‐Env containing K‐TM subunits are coactivated in T cell. However, these K‐TM subunits are not solely viral immune checkpoint (VIC) of cancer, a large number of other types of HERV‐TM subunits, such as H‐TM, F‐TM, and W‐TM might also be potential VIC cocktail effector. These data, together with the fact that high levels of glycosylated K‐TM subunits existed in sera of AML and PDAC patients and K‐TM exhibited high affinity binding to CD3ε and inhibits TCR signaling, imply that the immunosuppressive effect of HERV K‐TM in this process. Finally, we found that the K‐TM subunit is highly conserved. Targeting K‐TM could improve CD8^+^ T cell antitumor response by reversing CD8^+^ T cell anergy and restoring T cell‐mediated tumor cell killing.

Overall, our data support the new notion that the K‐TM subunit is a viral immune checkpoint that induces CD8^+^ T cell anergy and impairs T cell antitumor function via directly targeting CD3ε and blocking TCR‐signaling. Our findings provide novel targets and intervention strategies for cancer with immunotherapeutic resistance like PDAC and AML. Given that the suboptimal efficacy of PD‐1 blockade in immunologically “cold” tumors, combinatorial targeting of K‐TM with PD‐1 inhibitors would represent a promising translational strategy. We recognize current limitations in clinical translation and explicitly position the combinatorial targeting strategies as a priority research trajectory. Actually, we are currently generating K‐TM‐neutralizing monoclonal antibodies (patent‐pending), to enable such combination studies in our upcoming tumor immunotherapy research.

## 4 Experimental Section

### Cell Lines and Culture

Jurkat and MCA205 cells were cultured in RPMI 1640 (Invitrogen) supplemented with 10% FBS (Braserum) and 1% penicillin/streptomycin (TBD). MIA‐paca2, Hek293T, and KPC cells were maintained in DMEM (Invitrogen) with 10% FBS and 1% penicillin/streptomycin. Cells were grown in the humidified incubator containing 5% CO_2_ at 37 °C and routinely tested free of mycoplasma.

### CD8^+^ T Cell Isolation and Culture

Peripheral blood mononuclear cells (PBMCs) were separated by density gradient centrifugation using Lymphocyte Separation Medium (TBD Science, LTS1077). CD8^+^ T cells were positively selected from PBMCs using CD8 Nanobeads (GenScript, L00864). Cell purity was determined > 95% by flow cytometry. Isolated CD8^+^ T cells were cultured in IMDM (Cienry) containing 10% FBS (Gibco), 1% penicillin/streptomycin, IL‐2 (300U mL^−1^, QUANGANG PHARMACEUTICAL), IL‐7 and IL‐15 (5 ng mL^−1^, CRGEN). CD3/CD28 Dynabeads (Invitrogen, 11161D) were added for the activation and expansion of T cells for 5–7 days.

### Flow Cytometry

FCM was performed applying the NovoCyte flow Cytometer (ACEA, Biosciences, Inc.). Commercial antibodies for FCM were purchased and information was listed as below: CD45‐PB (clone HI30, Cat 304 022), CD3‐FITC (clone OKT3, Cat 317 306), CD8‐PE/Cy7 (clone SK1, Cat 344 712), CD4‐PE (clone RPA‐T4, Cat 300 507), CD107a‐APC (clone H4A3, Cat 328 619), Granzyme B‐APC (clone QA16A02, Cat 372 203), IFN‐γ‐PE/Cy7 (clone 4S.B3, Cat 560 741), PD‐1‐APC (clone EH12.2H7, Cat 329 907), Tim‐3‐APC/Cy7 (clone F38‐2E2, Cat 345 025), CD25‐PE/Cy7 (clone BC96, Cat 302 611), CD69‐APC/Cy7 (clone FN50, Cat 310 913). Antimouse‐CD45‐PB (clone 30‐F11, Cat 103 126), Antimouse‐CD3‐FITC (clone 17A2, Cat 100 204), Antimouse‐CD8a‐PE (clone 53–6.7, Cat 100 708), Antimouse‐CD107a‐APC (clone 1D4B, Cat 121 613), Antimouse‐Tim3‐PE/Cy7 (clone B8.2C12, Cat 134 009), Antimouse‐PD‐1‐APC/Cy7 (clone 29F.1A12, Cat 135 223), Antimouse IFN‐γ‐APC/Cy7 (clone XMG1.2, Cat 505 849), Antimouse‐Granzyme B‐APC (clone QA16A02, Cat 372 203).

For detecting the K‐Env expression, P236186, the specific viral K‐Env antibody were applied. P236186 antibody was screened from a natural human phage antibody library generated in our lab with K‐Env protein and synthesized with hFc‐tag. Its specificity was confirmed by FCM and ELISA, then used as the primary antibody with 1:200 dilution ratio. Goat F(ab')2 Anti‐Human IgG‐Fc‐PE (clone Poly23980, Cat 398 004) was used as the secondary antibody.

### Western Blot Analysis and Antibodies

Cells were treated and collected for lysis by M‐PER Mammalian Protein Extraction Reagent (Invitrogen, 78 501) containing the 1% protease and phosphatase inhibitor cocktail (Invitrogen, 78 428). Protein concentrations were quantified by Pierce BCA Protein Assay Kit (Invitrogen, 23 225). Equal‐quality proteins were subjected to SDS‐PAGE and transferred to PVDF membranes (BIO‐RAD, #1 620 177). Membranes were incubated with commercial primary antibodies and scanned by Tano 5200 Chemiluminescent Imaging System. Viral K‐Env antibody P233295 was prepared from a natural human phage antibody library and screened with K‐Env protein in the lab, and its dilution ratio for western blotting was 1:1000. Its specificity was confirmed by antigen blocking assay (Figure S2, Supporting Information). P233295 was pre‐incubated with K‐Env protein for immunization with molar 1:1 ratio of antigen/antibody at 4 °C overnight, and then applied for western blot detection.

Commercial antibodies involved in western blot assay were listed below. Primary antibodies: GAPDH (60004‐1‐Ig) and HIS (66005‐1‐Ig) from Proteintech; ZAP70 (ab32429), Lck (ab32149), ERK (ab184699), and Phos‐ERK (ab47339) from Abcam; Phos‐ZAP70 (2717), Phos‐Lck (2751), and HA (3724) from Cell Signaling Technology; Flag (F1804) from Sigma; pTyr (ET1704‐20) from Huabio; CD3ε (sc‐20047) from Santa Cruz; MMTV (100‐401‐P13) from Rockland Immunochemicals. Secondary antibodies: Goat anti‐Mouse IgG (HA1006), Goat anti‐Rabbit IgG (HA1001) from Huabio. All antibodies were certified according to the western blot images on their official website.

### Detection of K‐Env Expression in PDAC TILs

PDAC tumor tissues were shredded in complete DMEM medium, then placed in digestive solution containing 2% FBS, DNase I (100 µg mL^−1^, Solarbio life sciences), Collagenase IV (1 mg mL^−1^, Solarbio life sciences) and Hyaluronidase (0.1 mg mL^−1^, Solarbio life sciences), 37 °C, 200 rpm, digested for 2 h. The digested solution was filtered into 50 mL centrifuge tube using a 200‐mesh strainer and added with complete medium to terminate the digestion. After centrifuge for 2000 rpm, 10 min, the supernatant was discarded and the precipitation was resuspended with PBS. The prepared single‐cell tumor suspension was used for the subsequent FCM assay.

### Establishment of ELISA Kit and Detection of K‐Env in Sera

To detect K102 K‐Env protein levels in sera, the paired antibodies were employed, numbered A115 and A164, which were screened from the antibody library prepared by phage display technology in the lab, to establish a double‐antibody sandwich ELISA kit specifically targeting the K102 Env subtype. The detailed experimental steps were described below.

ELISA plates (Corning, 3690) were coated with 4 µg mL^−1^ A115 antibody in PBS, 30 µL per well, and incubated overnight at 4 °C. Next day, plates were washed with PBST for 3 times and blocked with 5% PBSM for 2 h at RT. Sera was added in 1:5 dilution, 30 µL per well, and incubated for 1 h at RT, while K102 Env antigen was added as the reference standard. Plates were washed with PBST 3 times and 4 µg mL^−1^ biotinylated A164 antibody was added, 30 µL per well for 1 h at RT, followed by NeutrAvidin‐HRP (Invitrogen, 31 001), 1:2000 in PBS, 30 µL per well for 50 min at RT. Plates were developed by adding 50 µL TMB substrate (Beyotime, P0209), followed by adding 50 µL of TMB stop solution (Beyotime, P0215). OD_450_ was measured on Spectramax Absorbance Reader (Molecular devices). The levels of K102 Env were quantified according to the assay's standard curve, and biological replicates were performed for each sample.

### Plasmids Construction and Transfection

Full‐length K‐TM amino acid sequence was originated from the transmembrane region (UniProtKB, Q69384.1, 439 ‐699 aa) of the retrovirus group K member 6 Env polyprotein (HERV‐K108, ERVK‐6, located on chromosome 7p22.1). K‐TM amino acid sequence, along with a signal peptide (UniProtKB, Q69384.1, 1‐89 aa) on the N‐terminal and HA‐tag on the C‐terminal, was reverse transcripted to nucleotide via codon optimization and cloned into the expression vector pcDNA3.1 (+) to construct pcDNA3.1‐K‐TM‐HA plasmid. K‐TM deletion mutants: K‐TM‐ΔTMD (1‐167 aa), K‐TM‐ΔFP (22‐234 aa), and K‐TM‐ΔFP+ISU (74‐234 aa) were designed and inserted into pcDNA3.1 (+) along with HA‐tag on the C‐terminal. Full‐length K‐TM‐HA was also inserted into the doxycycline‐inducible lentiviral expression vector pCW‐Cas9‐Puro (addgene) to generate pCW‐K‐TM‐HA plasmid. Complete coding region of human CD3ε (NM_000733) was inserted into pcDNA3.1‐Flag vector (YouBio, F116499) and truncated CD3ε including CD3ε‐ΔITAM (1‐184 aa), CD3ε‐ΔITAM+PRS (1‐177 aa), and CD3ε‐ΔITAM+PRS+BRS (1‐152 aa) were cloned into pcDNA3.1 with Flag‐tag on the C‐terminal. All plasmids were constructed via homologous recombination using Hieff Clone Plus One Step Cloning Kit (YEASEN, 10911ES20) and further verified by DNA sequencing (GenScript). In vitro DNA transfection reagent Polyjet (SignaGen, SL100688) was used with a 1:3 ratio of plasmid/Polyjet for plasmid transfection and cells were collected and lysed for various experiments after 48 h‐transfection.

### Lentivirus Package and Infection

Stable cell lines expressing target genes were established by lentivirus infection. Briefly, Hek293T cells were preseeded in 10 cm dishes to achieve 90% density and then transfected with a mixture of 9 µg target plasmids, 3 µg pMD2.G (Addgene, 12 259), 3 µg psPAX2 (Addgene, 12 260), and 45 µL Polyjet. The mixture was incubated in serum‐free DMEM medium at RT for 15 min, then added to Hek293T cells. Viral supernatants were collected separately at 24, 48, and 72 h after transfection, and filtered with 0.22 µm Ultra filters (Millipore, UFC910024) followed by super‐centrifugation at 4000 g for 2 h. Virus were added to host cells with 10 µg mL^−1^ polybrene (Santa Cruz, sc‐134220) and the positive clones were selected by growth in media with puromycin (MCE, HY‐K1057) or blasticidin (YEASEN, 60218ES10). Stable cell lines were generated after selection and the expression levels were determined by western blot.

### Cytotoxicity Assay

Luciferase assay was applied to detect the antitumor effects of CD8^+^ T cells. Luciferase‐expressing MIA‐paca2 tumor cells (5 × 10^4^) were cocultured with CD8^+^ T cells (Experiment group, Exp) at various E:T ratios in a 96‐well plate, and no T cells were added for the minimal cell killing group (Control). After 48 h incubation, plates were centrifuged at 1500 rpm for 5 min and supernatant was carefully discarded. Cells were added with D‐Luciferin salt bioluminescent substrate (1.5 mg mL^−1^, PerkinElmer), and measured by Specctramaxi3 instrument (Molecular Devices, Sunnyvale, USA). Lysis ability was calculated using the following formulas: Lysis ability = (1‐Exp/Control) × 100%, and each experiment was repeated at least 3 times.

Crystal violet staining assay was synchronously applied to evaluate the antitumor effects of PDAC CD8^+^ T cells against tumor cells. T cells were removed after 48 h incubation and the residual MIA‐paca2 tumor cells were fixed with 4% paraformaldehyde for 30 min followed by staining with crystal violet overnight. The next day, crystal dye was aspirated and washed with PBS for three times. Alive MIA‐paca2 cells were stained purple, and pictures were taken with OLYMPUS CKX53 microscope and manipulated with ToupView software. At least three visual fields per well were taken and the representative pictures were shown. Alive MIA‐paca2 cells were counted by Image J. Lysis ability was calculated using the following formulas: Lysis ability = (1‐alive cells_Exp_ /alive cells_Control_) × 100%, and each experiment was repeated at least 3 times.

### CD107a Assay

T cells and tumor cells were cocultured (E:T = 5:1) in 1640 medium, supplemented with 10% FBS and cytokines that included 300 IU mL^−1^ IL‐2, 5 ng mL^−1^ IL‐7, and 5 ng mL^−1^ IL‐15 for 5 h at 37 °C. GolgiStop protein transport inhibitor (Cat 51‐2092KZ, BD Bioscience) and CD107a‐APC antibody (clone H4A3, Cat 328 619) were added into the culture medium. After 5 h incubation, cells were labeled with CD8‐PE/Cy7 and analyzed by FCM. The percentage of CD107a^+^ cells was gated on live CD8^+^ T cells.

### K‐TM Expression Modulation: Knockout and Overexpression

To induce K‐TM silence in CD8^+^ T cells, single‐guide RNA was designed and synthesized by GenScript, sequence for K‐TM KO: 5′‐TGACAGGGTTAAGATTTGCG‐3′. T cells were centrifuge at 300 g for 5 min and washed with PBS, then resuspended in buffer at the desired concentration to obtain 1 × 10^6^ cells per reaction in a volume of 100 µL. RNP complexes were synchronously prepared: TrueCut Cas9 Protein v2 (60 pmol), and sgRNA (240 pmol) in a final volume of 10 µL of resuspension buffer per reaction. The mixture was incubated at RT for 10 min and added to cells, then pipetted into electroporation tip. 4D‐nucleofector Lonza was used and the program was set as EO‐115. To induce K‐TM silence in Jurkat T cells, single‐guide RNA was designed and inserted into pLX‐hPGK‐EGFP vector and constructed into lentivirus using the lentiviral packaging method described above. Transfection of sgRNA was performed with polyjet transfection reagent and cells were selected with puromycin (MCE, HY‐K1057) for 5 days and then used for cytotoxicity assay.

To induce K‐TM overexpression in CD8^+^ T cells, freshly isolated CD8^+^ T cells were maintained in nonactivated state through the CD3/CD28 deprivation. Cells were collected and washed with PBS, then mixed with pcDNA3.1 (+) vector carrying K‐TM gene and transferred into electroporation tip. 4D‐nucleofector Lonza was used and the program was set as EO‐115.

### Cell Proliferation Assay

Jurkat T cells and MCA205 cells stably overexpressed with pCW‐CTL (empty vector) or pCW‐K‐TM‐HA were established as described above. Cells in the logarithmic growth phase were preincubated with 1 µg mL^−1^ doxycycline to induce K‐TM expression and seeded in 96‐well plates (1.5 × 10^3^ cells per well) for a total volume of 200 µL. A total of 6 repetitive wells were set up, while a blank group was set up with only medium added. On day 0, 1, 3, 5, and 7, 20 µL thiazolyl blue tetrazolium bromide (MTT, Sangon Biotech, China) was added to each well at the same timepoint respectively. Cells were incubated with MTT in the incubator for 4 h and then dissolved overnight in a triple buffer containing 10% SDS, 5% isobutanol, and 0.012 m HCl. After the formazan crystals were completely dissolved, 96‐well plate was placed in a microplate reader and the OD value was determined at a wavelength of 562 nm. OD value of the experimental group was subtracted of the blank group, and cell growth curves were drawn by GraphPad Prism 9.0.

### T Cell Inhibition Assay

CD8^+^ T cells were treated with nonglycosylated K‐TM protein purified from the prokaryotic expression system at different concentrations (0, 5, 10, 50, and 100 µg mL^−1^) or glycosylated K‐TM protein purified from the eukaryotic expression system at different concentrations (0, 0.001, 0.01, 0.1, 1, and 5 µg mL^−1^), then activated with CD3/CD28 dynabeads for 7 days. T cell proliferation level was measured with the MTT assay and the growth inhibition rate was calculated by the follows: (1‐OD_K‐TM_/OD_control_) × 100%. To monitor the effect of glycosylated K‐TM protein on CD8^+^ T cell proliferation in microscope, CD8^+^ T cells were isolated and divided equally into two groups: one was pretreated with 1 ng mL^−1^ glycosylated K‐TM protein for 12 h, and another with PBS as the negative control. Both two groups were cultured with CD3/CD28 dynabeads to stimulate T cell activation. At different time points on day 1, day 3, day 5, and day 7, cell proliferation was monitored and images were taken by microscopy.

### IFN‐γ and TNF‐α Cytokine Detection

At the endpoint of day 7 of T cell inhibition assay, cell debris was removed by centrifugation at 1000 g for 5 min and the supernatant was harvested to measure cytokine release including IFN‐γ and TNF‐α employing Human Th1/Th2/Th17 Cytokine Kit (CEGER). Data were analyzed using FCAP Array Software Version 3.0 (BD Biosciences) following the manufacturer's instructions.

### CFSE Assay

Freshly isolated CD8^+^ T cells were maintained in nonactivated state through the CD3/CD28 deprivation and preincubated with varying concentrations of glycosylated and nonglycosylated K‐TM proteins for 12 h. Cells were labeled with Carboxyfluorescein Diacetate Succinimidyl Ester (CFSE) cell proliferation kit (TargetMol, T6802) and cultured in T cell complete medium, then collected at different indicated timepoints and detect by flow cytometry to analyze T cell growth.

### Co‐immunoprecipitation Assay

To determine the interaction of CD3ε between K‐TM and its truncated mutants, Hek293T cells were cotransfected with HA‐tagged K‐TM (WT or truncated K‐TM, ΔTMD, ΔFP, ΔFP+ISU) and WT CD3ε. After transfection for 48 h, cells were lysed in NP‐40 buffer including 1% EDTA and 1% protease inhibitor cocktail for 30 min followed by centrifuge of 15 000 g for 15 min. Cell supernatant was incubated with anti‐HA magnetic beads (Bimake, B26201) at 4 °C overnight. For interaction of K‐TM between CD3ε and its truncated mutants, Hek293T cells were cotransfected with Flag‐tagged CD3ε (WT or truncated CD3ε, ΔITAM, ΔITAM+PRS, ΔITAM+PRS+BRS) and WT K‐TM, cell lysates were combined with anti‐Flag magnetic beads (MCE, HY‐K0207). Beads were washed with NP‐40 lysis buffer containing 0.1% Tween‐20 for 8 times and eluted with 2 × loading sample buffer at 100 °C for 10 min. Supernatant was subjected to western blot.

For endogenous CD3ε co‐IP in Jurkat T cells, cells expressing K‐TM‐HA were cultured in the presence of 1 µg mL^−1^ doxycycline for 48 h, then collected and lysed in M‐per buffer containing 1% EDTA, 1% protease and phosphatase inhibitor cocktail, 1% digitonin for 1 h. Lysates were divided into two tubes and incubated with IgG isotype (HUABIO, HA1002) or HA antibody (CST, 3724) at 4 °C overnight on a rotator. The next day, protein A/G magnetic beads (Invitrogen, 26 162) were added to each sample, and the mixture was further rotated for 6 h at 4 °C. Beads were washed and eluted as described above.

For MMTV‐env co‐IP in KPC cells, HA labeled hFL protein was used. KPC cells were lysed in lysis buffer for 30 min followed by centrifuge of 15 000 g for 15 min. Cell supernatant was divided and incubated with IgG isotype or 100 µg mL^−1^ hFL protein at 4 °C overnight. Protein A/G magnetic beads (Invitrogen, 26 162) were added to each sample and incubated for 6 h at 4 °C. Beads were washed and eluted. Immunoprecipitated protein was subjected to WB and detected by MMTV primary antibody.

For K‐TM and hFL co‐IP, Hek293T cells were co‐overexpressed with HA‐tagged K‐TM and His‐tagged hFL. Cell lysates were collected and precipitated by His antibody (Proteintech, 66005‐1‐Ig), then incubated with protein A/G beads. Beads were washed and eluted as described above.

### Immunofluorescence

For detection of soluble K‐TM protein and CD3ε colocalization, mCherry‐tagged K‐TM protein were prepared and freshly isolated CD8^+^ T cells were maintained in nonactivated state through CD3/CD28 deprivation. Cells were treated with soluble K‐TM protein, then seeded on coverslips and fixed in 4% paraformaldehyde. Coverslips were coincubated with CD3ε primary antibody (Abcam, ab16669) in blocking buffer at 4 °C overnight, then stained with Alexa Fluor 488 anti‐mouse secondary antibodies (YEASEN, 34106ES60) for 1 h at RT. For detection of K‐TM and hFL colocalization, Hek293T cells were transfected with pLVX‐K‐TM‐mCherry for 48 h. Cells were grew in confocal dishes and treated with 10 µg mL^−1^ HA labeled hFL protein for 6 h. Then cells were washed and incubated with HA antibody in blocking buffer at 4 °C overnight and stained with Alexa Fluor 488 anti‐mouse secondary antibody for 1 h at RT. Nucleus were stained with DAPI for 10 min and imaged with Zeiss Confocal Laser Scanning Microscope 710 (LSM710, Germany). ZEN software was used for image acquisition and analysis.

### Bio‐layer Interferometry

Bio‐layer interferometry (BLI) detecting the protein affinity was conducted using Octet R8 (Sartorius BioAnalytical Instruments Inc.) by Acnovia Biosystems (Hangzhou). Protein was immobilized on an SA (Sartorius) chip through biotin conjugation in immobilization of 600 s resulting immobilization signal of 4.5 nm. Tested protein was added to the plate with a twofold gradient dilution of PBST, the association time was set to 200 s and the dissociation time to 300 s. Data were analyzed by the fortebio data analysis 12.0 (Sartorius) and the affinity was represented by *K*_D_ value (nM).

### Phosphorylation Level Detection of CD3ε

Jurkat cells expressing pCW‐CTL or pCW‐K‐TM‐HA plasmids were treated with 1 µg mL^−1^ doxycycline for 48 h to induce K‐TM expression. Cells were harvested and lysed for 1 h, and cell debris were removed by centrifugation at 15 000 g for 20 min. Supernatant was incubated with CD3ε antibody at 4 °C overnight on a rotator. Next day, protein A/G magnetic beads (Invitrogen, 26 162) were added to the sample mixture and further incubated for 6 h at 4 °C. Beads were washed with 0.2% M‐Per lysis buffer for 8 times, then boiled with 2 × loading buffer at 100 °C for 10 min. Beads were removed with a magnet, supernatant was collected and subjected to WB for detection of the CD3ε phosphorylation.

### Binding Model of K‐TM with CD3ε

The binding model of K‐TM in complex of CD3ε was studied in HDOCK applying docking methods. The homology model of monomer CD3ε in the range of 153–207 aa was built by Swiss‐Model (https://swissmodel.expasy.org/), and the model of K‐TM protein was built in the range of 466–699 aa by AlphaFold2. The molecular dynamics simulation for the complex was studied by Gromacs2020 and calculation of binding energy was conducted by g_MMPBSA method. Pymol software was involved in exhibiting visual analysis of protein–protein interactions.

### MCA205 Tumor Rejection In Vivo Assay

C57BL mice‐derived MCA205 cells were overexpressed with inducible pCW vector or pCW‐K‐TM‐HA plasmids. Constructed MCA205‐CTL and MCA205‐K‐TM‐HA cells (1 × 10^6^ per mouse) were injected subcutaneously in the unilateral flank of 6‐week‐old BALB/c mice (SLAC ANIMAL). To induce K‐TM expression, mice were administrated with 2 mg kg^−1^ doxycycline per day. Tumor growth was monitored with vernier calipers twice weekly and tumor volume (mm^3^) was determined by measuring the tumor width and length. Tumor volume was calculated using the formula: (length × width^2^)/2. Mice were given euthanasia after 20 days and tumors were taken for analysis of tumor weight, western blot, and H&E staining. The IS indexes of K‐TM were calculated as (A_K‐TM_‐A_CTL_)/A_CTL_, where A_K‐TM_ and A_CTL_ are the mean areas at the peak of growth of tumors from BALB/c mice injected with MCA205 cells expressing K‐TM gene or with empty vectors, respectively. For Histopathology, tumors were fixed in 4% paraformaldehyde, dehydrated, and embedded in paraffin, sectioned and processed for H&E staining. For immunofluorescence staining, tumor slides were stained with CD8‐Cyanine3 and representative pictures were taken with Olivia software. For quantification of infiltrated CD107a^+^ CD8^+^ T cell levels and the percentages of Granzyme B^+^, IFN‐γ^+^, Tim‐3^+^, PD‐1^+^ cells within tumor‐infiltrating CD8^+^ T cells, tumors were digested into single‐cell suspensions for FCM.

### KPC Tumor Growth Inhibition In Vivo Assay

KPC cells harboring mutant KRA_SG12D_ and TP53_R172H_ were overexpressed with pCDH‐luciferase plasmids to develop Luciferase‐tagged KPC cells (KPC‐Luci). KPC‐Luci (1 × 10^6^ per mouse) were implanted subcutaneously in the left flank of 6‐week‐old C57BL mice (Cyagen). Since tumor signals could be detected, mice were randomized into two groups to receive water or hFL lectin (5.0 mg kg^−1^) via intravenous injections (i.v) every 2 days. Tumor burden was monitored by IVIS lumina LT series III in vivo imaging system (Perkin Elmer, USA), and tumor volume was measured synchronously every 5 days.

### Ethical Statement

Primary peripheral blood samples including AML, PDAC, and normal subjects, and tumor tissues from PDAC patients were obtained from clinical residual blood samples with the waiver of informed consent from ethics approval (2022‐0791). All experimental process was conducted under the permission of the Ethics and Scientific Committee of The Second Affiliated Hospital of Zhejiang University School of Medicine. The animal experiments were approved by the ethics committee of The Second Affiliated Hospital of Zhejiang University School of Medicine with the approval number 2023–152. The work was approved by The Second Affiliated Hospital of Zhejiang University Ethics Committee.

### Statistical Analysis

Statistical analyses were performed with GraphPad Prism 9.0, and results were presented as mean ± s.e.m. Statistical analyses were performed using two‐tailed student's *t*‐test, two‐way ANOVA, and Mann–Whitney test, while the data were not in line with the Gaussian distribution. *p* < 0.05 was considered to be statistically significant, the difference was shown as follows: * *p* < 0.05, ** *p* < 0.01, *** *p* < 0.001, **** *p* < 0.0001, and ns *p* > 0.05.

## Conflict of Interest

The authors declare no conflict of interest.

## Author Contributions

M.L., S.Z., Q.G., Z.W., and W.L. contributed equally to this work. R.Z.X. conceived the study, initiated, designed, supervised the experiments, R.Z.X. and M.Y.L. wrote the manuscript. M.Y.L., S.W.Z., Q.Y.G., Z.X.W., W.L., W.Y.C., P.W., X.Z.Z., Y.L., and F.L.L. performed experiments. W.B.Q., Y.L., and Q.Z. supervised the experiments.

## Supporting information



Supporting Information

## Data Availability

The data that support the findings of this study are available from the corresponding author upon reasonable request.
