# Supplementary material for: HERV‐K TM Subunit Elicits CD8+ T Cell Anergy and Tumor Immune Evasion via Targeting CD3 Coreceptor ε in AML and PDAC
Source: Adv Sci (Weinh). 2025 Aug 13;13(1):e17432. doi: 10.1002/advs.202417432 (PMC12767058; doi:10.1002/advs.202417432)
Supplement: Supplementary file 1 — Supporting Information [file ADVS-13-e17432-s001.docx]

Supplementary Information

HERV-K TM subunit elicits CD8^+^ T cell anergy and tumor immune evasion via targeting CD3 coreceptor ε in AML and PDAC

*Mengyuan Li^1,2,7^, Shuwen Zheng^1,2,7^, Qinyuan Gong^1,2,7^, Zhaoxing Wu^1,2,7^, Wen Lei^1,7^, Wanyue Cao^3,4,5^, Ping Wang^1^, Xuzhao Zhang^1^, Wenbin Qian^1,2^, Yun Liang^1^, Ying Lu^6^, Fenglin Li^6^, Qi Zhang^3,4,5*^, Rongzhen Xu^1,2*‡^*

^1^Department of Hematology and Cancer Institute (Key Laboratory of Cancer Prevention and Intervention, China National Ministry of Education), the Second Affiliated Hospital, Zhejiang University School of Medicine, Hangzhou 310009, China.

^2^ Institute of Hematology, Zhejiang University, Hangzhou, 310009, China.

^3^ Department of Hepatobiliary and Pancreatic Surgery, the First Affiliated Hospital, Zhejiang University School of Medicine, Hangzhou, 310009, China.

^4^ Zhejiang Provincial Key Laboratory of Pancreatic Disease, the First Affiliated Hospital, Zhejiang University School of Medicine, Hangzhou, 310009, China.

^5^ MOE Joint International Research Laboratory of Pancreatic Diseases, the First Affiliated Hospital, Zhejiang University School of Medicine, Hangzhou, 310009, China.

^6^ Department of Hematology, the Affiliated People's Hospital of Ningbo University, Ningbo, 315000, China.

^7^ These authors contributed equally to this work.

* Corresponding authors.

^‡^ Leading corresponding Author. Dr. Rongzhen Xu. Department of Hematology, The Second Affiliated Hospital, Zhejiang University School of Medicine, Hangzhou, Zhejiang, 310009, China. Email: [zrxyk10@zju.edu.cn](mailto:zrxyk10@zju.edu.cn)

Supplementary Experimental Methods

***Cell apoptosis detection***

Apoptosis of Jurkat-CTL and Jurkat-K-TM cells were detected applying Annexin V-APC/7-AAD apoptosis kit (Multi Sciences, AP105). Briefly, cells overexpressed with pCW-vector or K-TM were harvested after 7 days of doxycycline induction and washed twice with cold PBS, then stained with Annexin V-APC/7-AAD in 1×Binding Buffer away from light for 5 min. Data were detected by NovoCyte flow Cytometer and analyzed in FlowJo 10.8.1 software.

***Jurkat T cell anti-tumor assay***

Jurkat cells were pre-activated with 1×PMA/ionomycin cell stimulation cocktail (Invitrogen, 00-4970-03) for 12 h before co-cultured with MIA-paca2 expressing GFP tag with E/T ratio of 2:1, 3:1 and 5:1, separately. After 48 h incubation, GFP fluorescent intensity was detected as previously described and the remaining surviving MIA-paca2 cells were quantified.

***Co-immunoprecipitation assay***

To determine the interaction of CD3ε between K-TM and its truncated mutants, Hek293T cells were co-transfected with HA-tagged K-TM (WT or truncated domains of K-TM, ΔTMD, ΔFP, ΔFP+ISU) and WT CD3ε. At 48 h transfection, cells were lysed in buffer including 1 % NP-40, 1 % EDTA, and 1 % protease inhibitor cocktail for 30 min followed by a centrifuge of 15000 g for 15 min. Cell supernatant was incubated with anti-HA magnetic beads (Bimake, B26201) at 4 °C overnight. For the interaction of K-TM between CD3ε and its truncated mutants, Hek293T cells were co-transfected with Flag-tagged CD3ε (WT or truncated domains of CD3ε, ΔITAM, ΔITAM+PRS, ΔITAM+PRS+BRS) and WT K-TM, cell lysates were combined with anti-Flag beads (MCE, HY-K0207). Beads were washed with NP-40 buffer containing 0.1% Tween-20 and eluted with 2×loading buffer at 100 °C for 10 min. Supernatant was subjected to western blot.

For endogenous CD3ε co-IP in Jurkat T cells, cells expressing K-TM-HA were cultured in the presence of 1μg mL^-1^ doxycycline for 48 h, then collected and lysed in a solubilization M-per buffer including 1% EDTA, 1% protease and phosphatase inhibitor cocktail, 1% digitonin for 1 h. Lysates were divided into two tubes and incubated with rabbit IgG (HUABIO, HA1002) or HA antibody (CST, 3724) at 4 °C overnight on a rotator. The next day, protein A/G magnetic beads (Invitrogen, 26162) were added to each sample, and mixture was further rotated for 6 h at 4 °C. Beads were washed and eluted as described above.

***Immunofluorescence***

Jurkat T cells were stably expressed with pLVX-K-TM-AcGFP plasmids or incubated with mCherry-tagged K-TM protein, GFP-tagged FP protein. Cells were seeded on coverslips and fixed in 4 % paraformaldehyde, then blocked with 5 % serum. Coverslips were incubated with CD3ε primary antibodies, then stained with Alexa Fluor secondary antibody. Slides were stained with DAPI for 10 min and imaged with Zeiss Confocal Laser Scanning Microscope 710 (LSM710, Germany). ZEN software was used for image acquisition and analysis.

***Eukaryotic protein expression and purification***

CHO Mammalian eukaryotic expression system and His-Ni immobilized metal ion affinity chromatography (Ni-IMAC) were used to purify K-TM and CD3ε protein. Briefly, CHO cells were transfected with constructed pcDNA3.1-K-TM-His or pcDNA3.1-CD3ε-His plasmids. After 72 h transfection, cell lysates were incubated with His-tag Purification Resin (Beyotime, P2229S) and subjected to affinity chromatography empty column (Beyotime, FCL12) on a rotator for 1 h at 4 °C. Column was washed with nondenaturing washing buffer and eluted with nondenaturing elution buffer. The eluted protein was concentrated by ultrafiltration and buffer was exchanged with PBS. Protein purity was monitored by SDS-PAGE and Coomassie Blue Staining.

For the expression and purification of hFL eukaryotic protein, hFL sequence was cloned into the pCGS3 vector to generate hFL-pCGS3 plasmid. CHO cells were stably expressed with hFL via electroporation, and 1 L of culture supernatant was obtained. Supernatant was flowed away protein A/G chromatography column, washed, and eluted to obtain purified hFL protein.

***Prokaryotic protein expression and purification***

For the expression and purification of prokaryotic K-TM and CD3ε protein, sequences of K-TM and CD3ε were inserted into pET41a and pSmart1 vectors, respectively, to generate plasmid pET41a-K-TM-His and pSmart1-CD3ε-GST. Plasmids were expressed in Rosetta (DE3) cells. Expression of the recombinant protein was induced by 1.0 mM IPTG at 37 °C. After 4 h induction, cells were collected by centrifugation and then smudged by sonication. Supernatant of lysates was purified by His or GST affinity chromatography. Protein was dissolved in PBS and purity was determined by SDS-PAGE.

***Identification and isolation of human sera with K-TM-reactive antibodies***

To identify positive sera containing high titer of K-TM-reactive antibody, we applied ELISA assay with K-TM antigen. Peripheral blood was collected from immune-hyperactive systemic lupus erythematosus (SLE) patients and normal subjects, then centrifuged to isolate sera. ELISA plates (Corning, 3690) were coated with purified K-TM antigen per well and incubate overnight at 4 °C. Next day, plates were washed with PBST for 3 times and blocked with 5 % PBSM for 2 h at RT. Sera ware added with different dilutions and incubated for 60 min at RT. Plates were washed with PBST for 3 times, anti-human-IgG-Fc-HRP (Huabio, HA1018) were added and incubated for 60 min at RT. Plates were developed with 50 μL TMB substrate (Beyotime, P0209), and stopped with TMB stop solution (Beyotime, P0215). OD_450_ was measured on Spectramax Absorbance Reader (Molecular devices).

***Conservative analysis of K-TM***

Candidate open reading frames (ORFs) were identified by performing tBLASTn search using K-TM amino acid sequences ranging from 466-699 aa. Amino acid sequences of the 67 candidates were aligned with K-TM using ggmsa-R language.


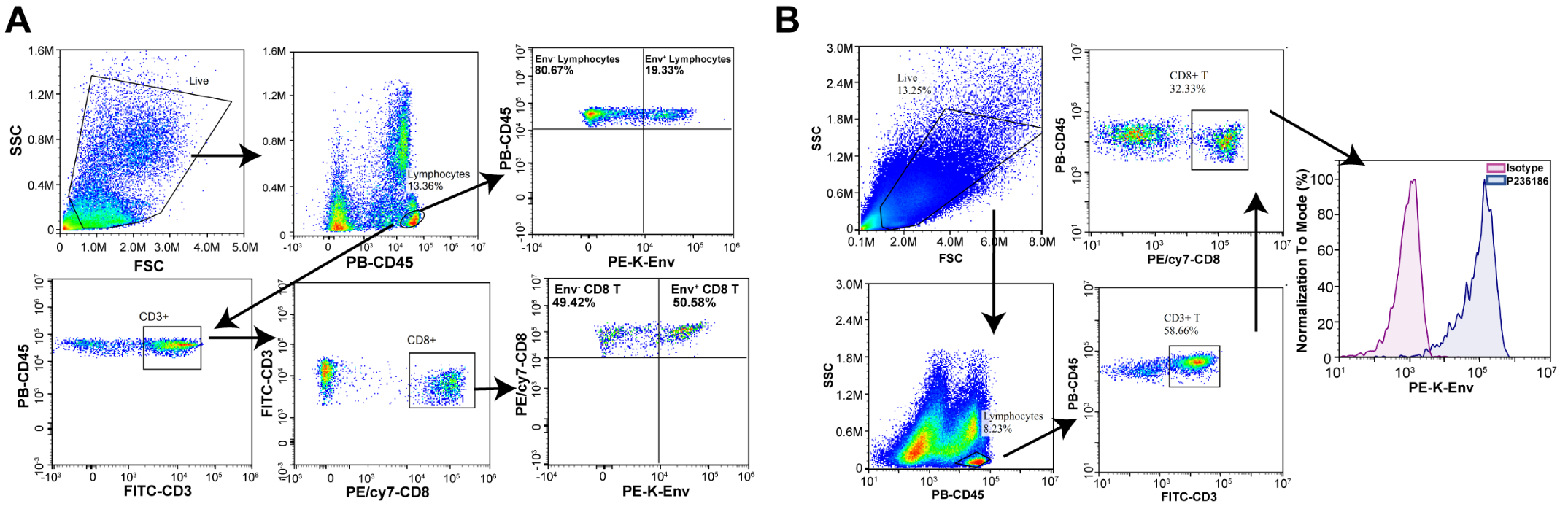


Figure S1. Flow cytometry gating strategy for detection of K-Env expression level in human primary samples

(**A**) Representative flow cytometry plots of the gating strategy used to determine K-Env protein expression on lymphocytes and CD8^+^ T cells from primary sample. (**B**) Flow cytometry plots of the gating strategy used to determine K-Env expression on tumor-infiltrating cells (TILs) as well as tumor-infiltrating CD8^+^ T cells from PDAC tissue.


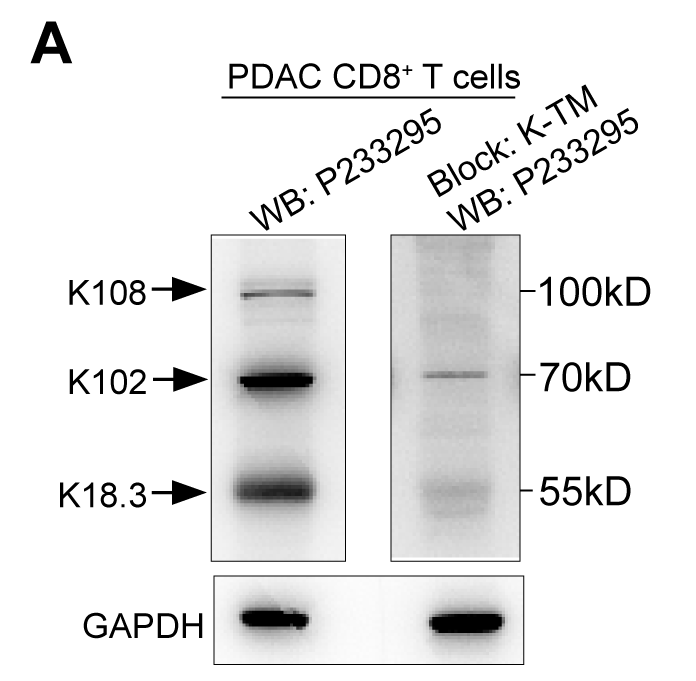


Figure S2. Specificity of P233295 antibody

(**A**) P233295 antibody specifically recognized endogenous K-Env proteins, including K108, K102, K18.3 subtypes in CD8^+^ T cells of PDAC patients (left). K-TM blocked the binding of P233295 antibody to endogenous K-Env proteins (right). GAPDH were stained as an internal control.


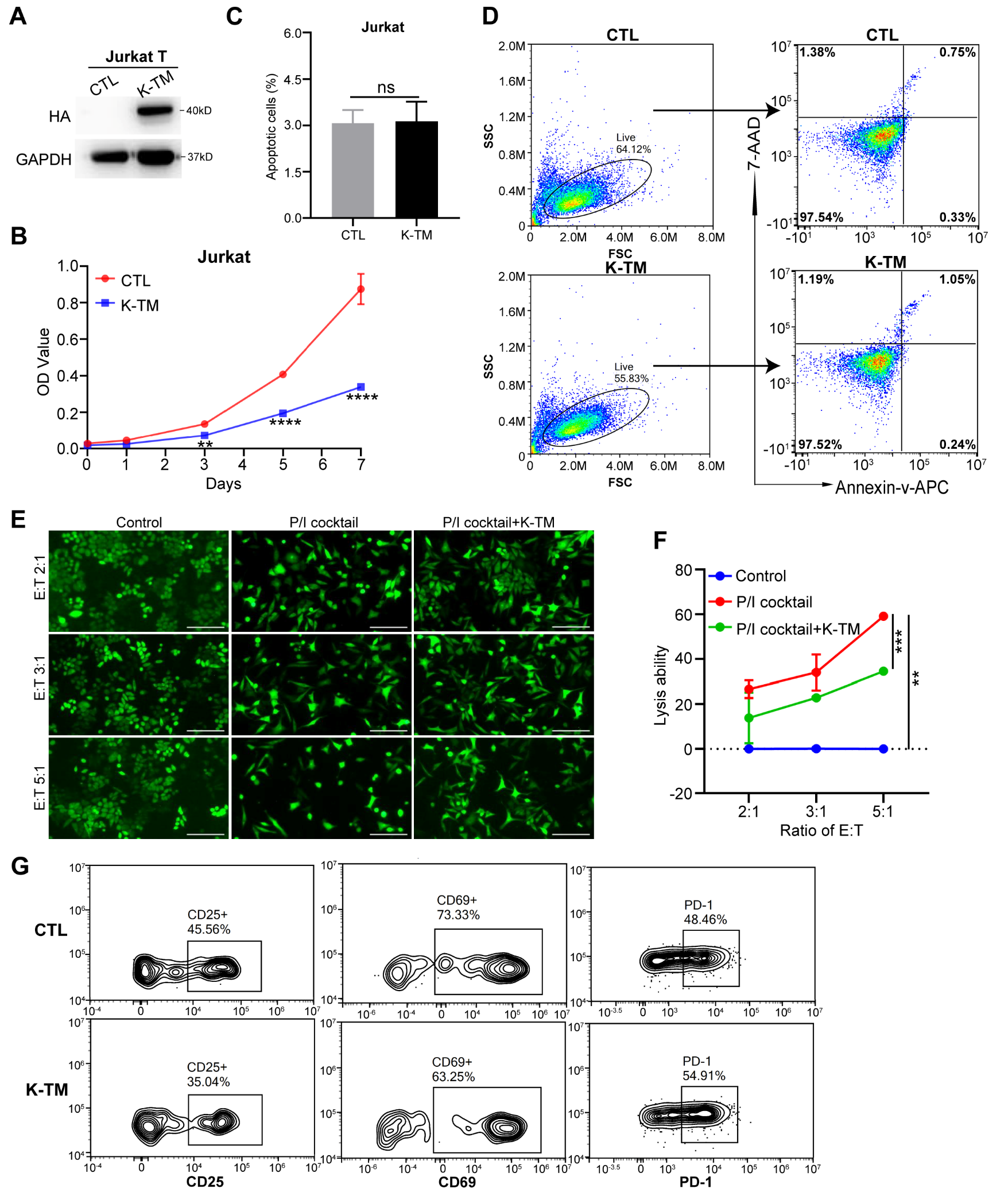


Figure S3. K-TM expression inhibits CD8^+^ T cell proliferation and anti-tumor function

(**A**) Western blot analysis of K-TM overexpression in Jurkat T cells. (**B**) Comparisons of Jurkat cell proliferation activity among K-TM or empty vector, assessed by MTT (n=3). (**C**) Apoptosis analysis in Jurkat cells expressing K-TM or CTL by flow cytometry. Results were repeated for three times. (**D**) Flow cytometry plots of the gating strategy used to detect the apoptosis of Jurkat cells. (**E**) K-TM reduced the anti-tumor function of P/I cocktail-activated in K-TM-high overexpressing Jurkat T cells. Results were representative of three biological tests. (**F**) Quantification of lysis ability among Jurkat T cells after indicated treatment. (**G**) FCM analysis was used for detecting the changes of activation-related molecule CD25 (left), CD69 (medium) and exhaustion-related molecule PD-1 (right) on CD8^+^ gated cells after non-glycosylated K-TM protein treatment. Statistical analyses: Error bars represent means ± s.d. Two-tailed unpaired t-test, **P< 0.01; ***P< 0.001; ****P< 0.0001 and ns P > 0.05.


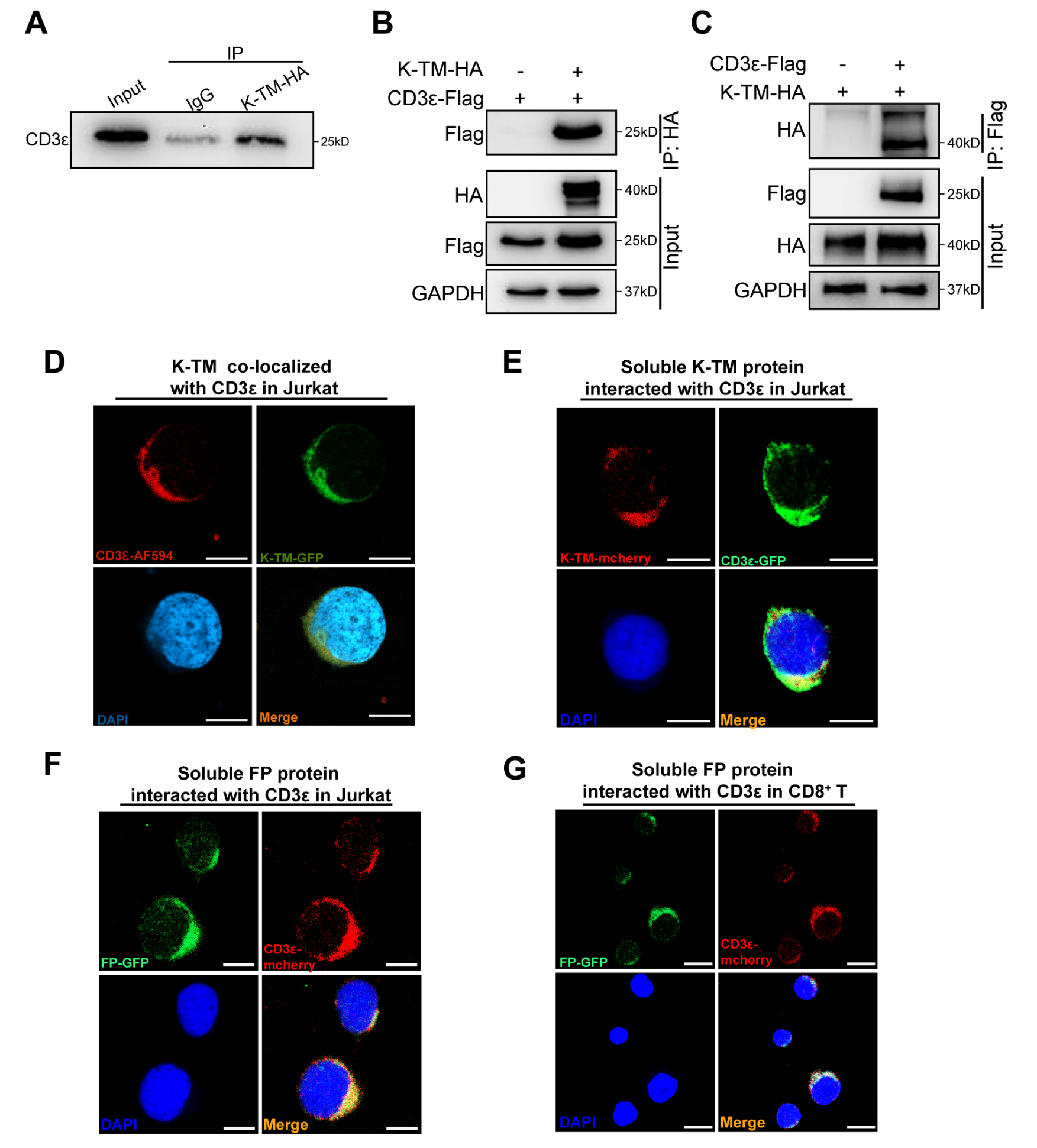


Figure S4. K-TM interacts with CD3ε coreceptor

(**A**) Western blot analysis for endogenous CD3ε protein in Jurkat T cells after K-TM-HA or IgG (negative control) co-IP assay. (**B**) Western blot analysis for exogenous CD3ε protein in Hek293T cells after K-TM-HA or IgG (negative control) co-IP assay. (**C**) Western blot analysis for exogenous K-TM protein in Hek293T cells after CD3ε-Flag or IgG (negative control) co-IP assay. (**D**) Colocalization analysis of endogenous CD3ε (red) with K-TM-GFP protein (green) in Jurkat T cells. Scale bars: 10 μm. (**E**) Co-localization analysis of soluble K-TM-mCherry (red) interacted with CD3ε-GFP (green) on Jurkat T cell membrane. Scale bars: 10 μm. (**F-G**) Co-localization analysis of soluble FP-GFP (green) interacted with CD3ε-mCherry (red) on Jurkat (F) and primary CD8^+^ T cell (G) membrane. Scale bars: 10 μm.


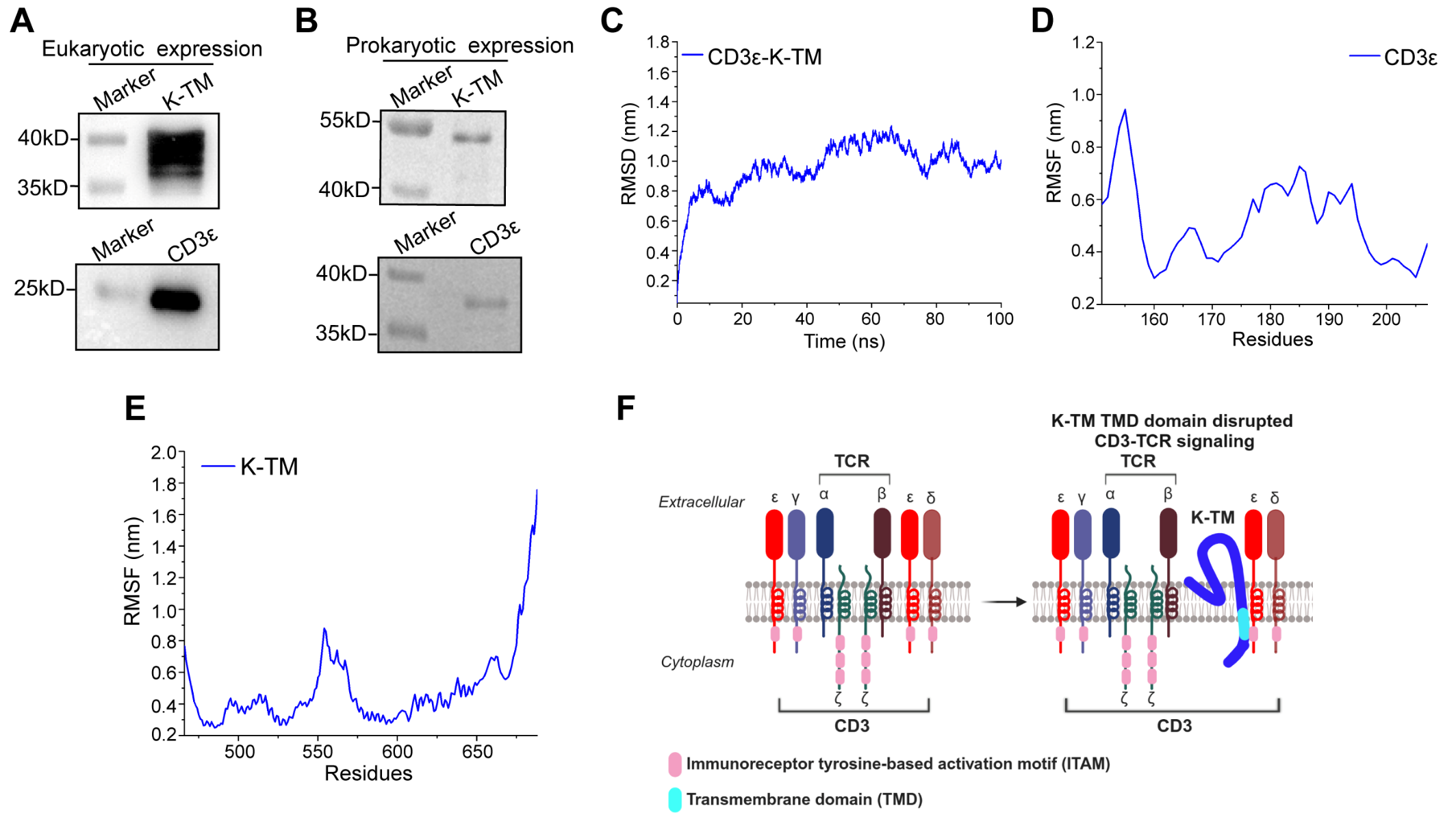


Figure S5. K-TM binds to the ITAM of CD3ε coreceptor via its TMD directly.

(**A** and **B**) Generation and western blot analysis of purified CD3ε protein and K-TM protein from eukaryotic expression system (**A**) and prokaryotic expression system (**B**). (**C**) RMSD plot during molecular dynamics simulations for the CD3ε-K-TM complex. (**D** and **E**) RMSF plot during molecular dynamics simulations for CD3ε (**D**) and K-TM (**E**) protein. (**F**) Scheme showing K-TM interacts with CD3ε ITAM domain via K-TM TMD and disables the TCR signaling by blocking CD3ε ITAM. Created with BioRender.com.


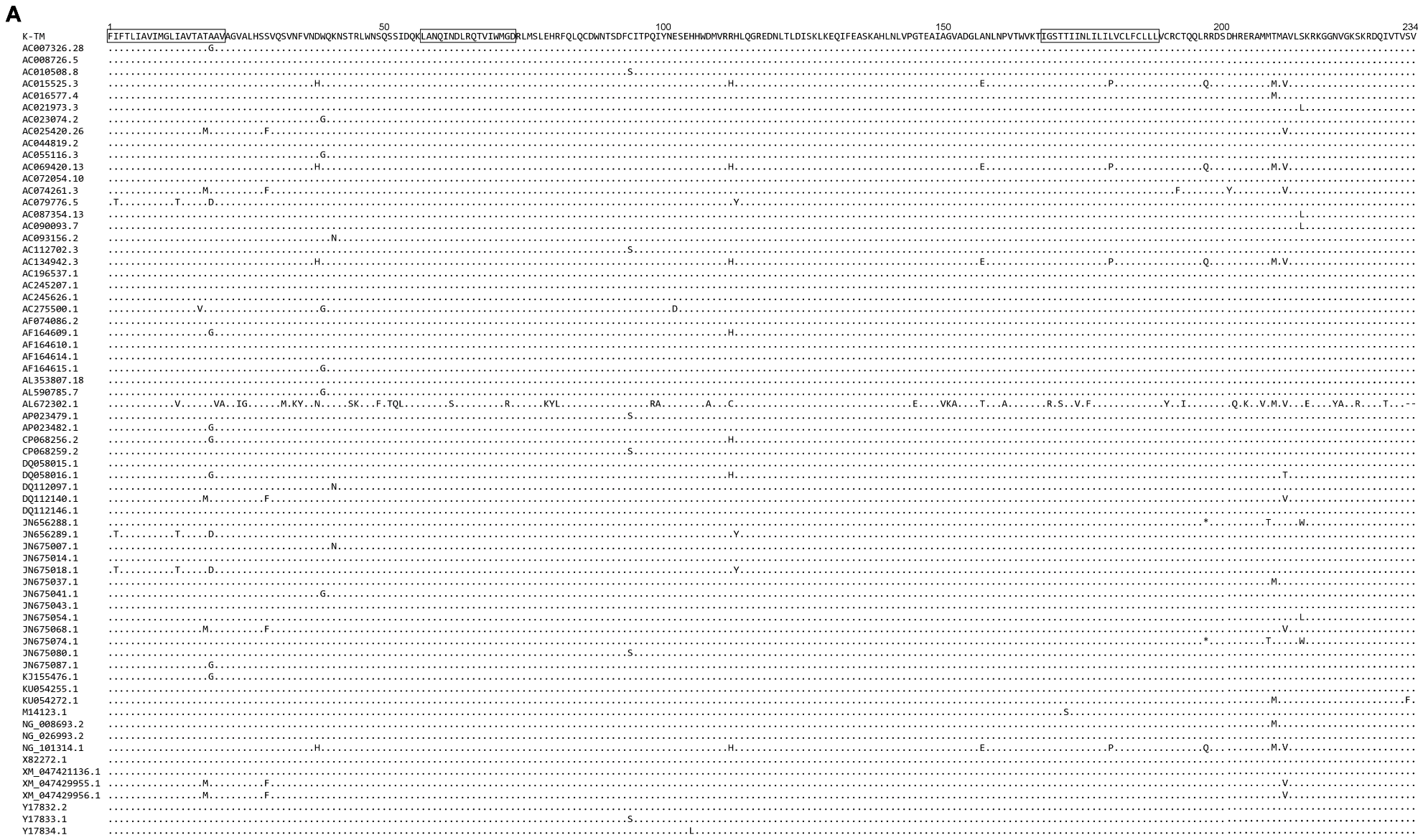


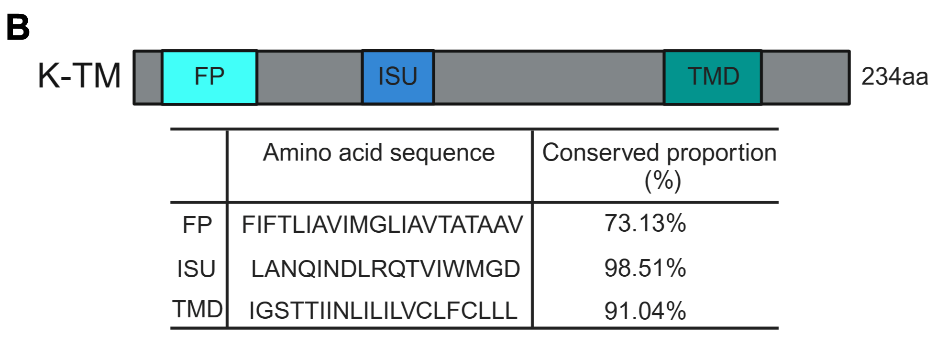


Figure S6. K-TM protein is highly conserved in the human genome

(**A**) Sequence alignment analysis of 67 intact ORFs for K-TM protein from GenBank. FP, ISU, and TMD domains were marked with blank rectangles. (**B**) Structure of K-TM and the proportion of FP, ISU, and TMD domains.


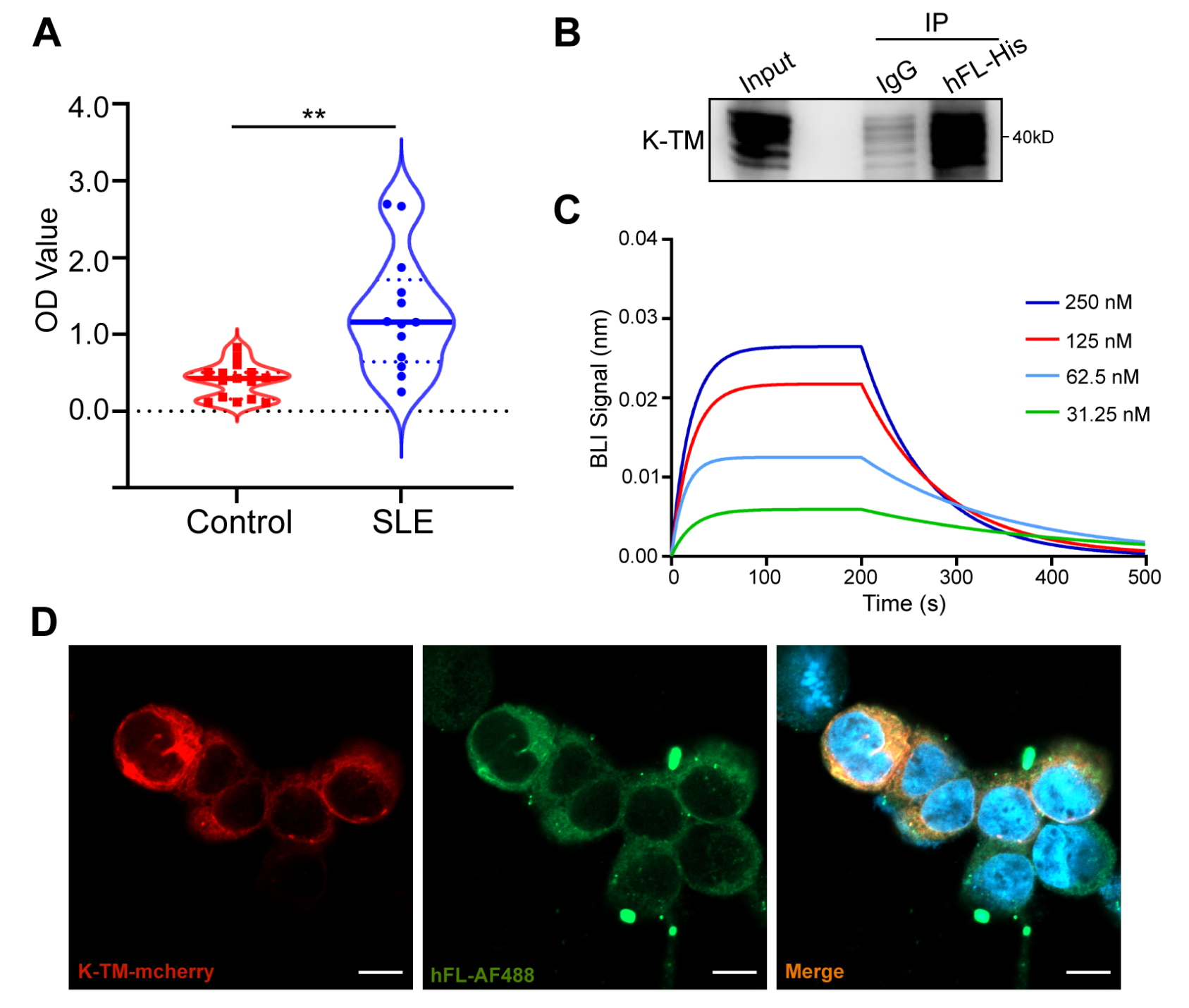


Figure S7. Human sera with K-TM-reactive antibodies and hFL lectin specifically targets K-TM protein

(**A**) ELISA analysis of K-TM-reactive antibody titers in sera from SLE patients (n=13), dilution ratio was 1:1000 and sera from normal subjects (n=15) were applied as control. Each sample was measured with two biological replicates and the average concentration was represented as mean ± s.e.m., P values were calculated by two-sided student’s t-test and **P< 0.01. (**B**) Western blot analysis for K-TM protein after IgG (negative control) or hFL co-IP assay. (**C**) Bio-layer interferometry (BLI) results showed the binding of K-TM to hFL protein in a dose-dependent manner. (**D**) Colocalization analysis of hFL-Alexa Fluor 488 (green) with K-TM-mCherry protein (red) in HEK293T cells after treatment with 10 μg mL^-1^ hFL for 6h. Scale bars: 10 μm.


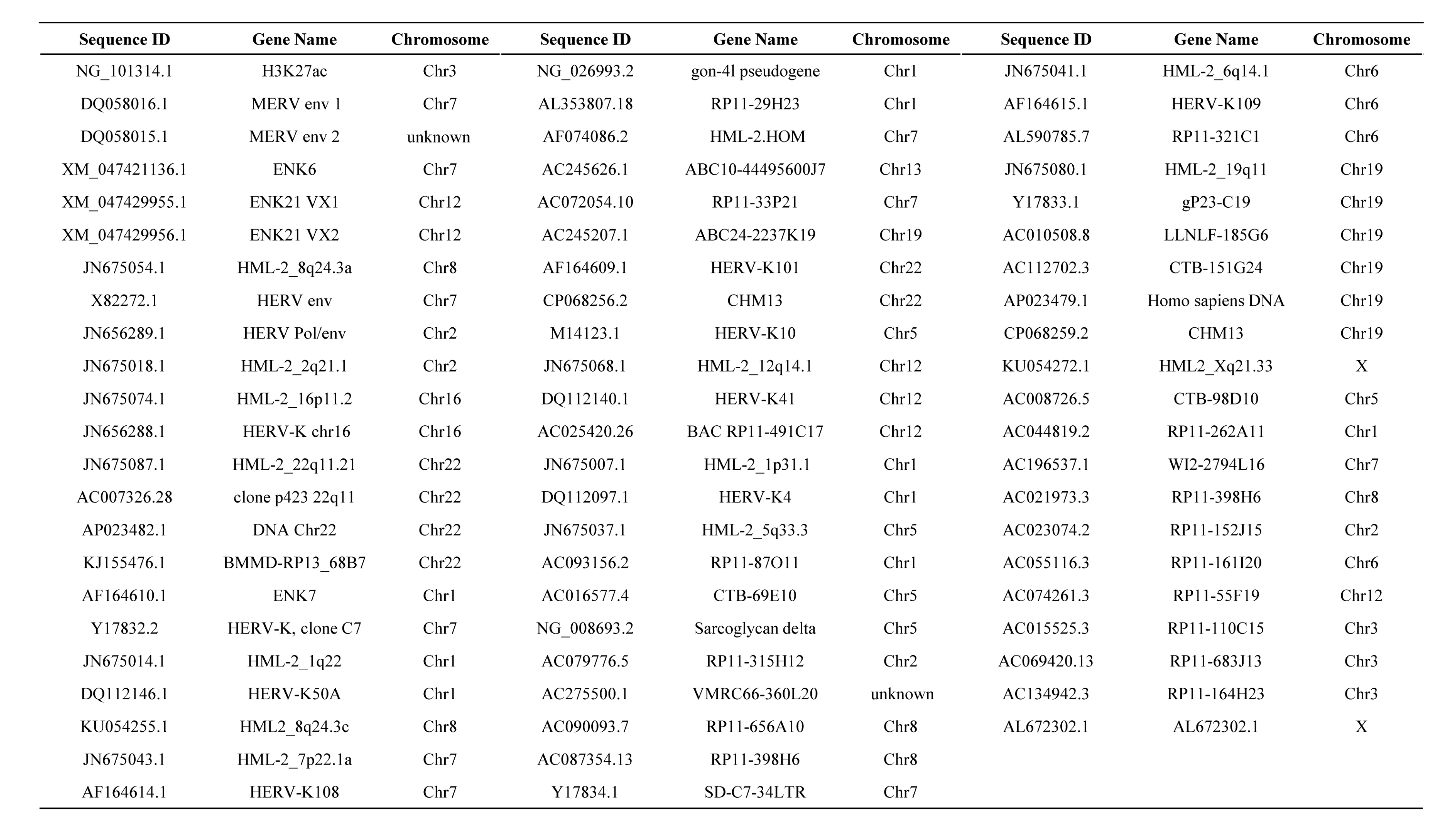


Supplementary Table 1. Gene name and chromosome location of 67 conserved ORFs in the human genome
